# MicroHDF: predicting host phenotypes with metagenomic data using a deep forest-based framework

**DOI:** 10.1093/bib/bbae530

**Published:** 2024-10-24

**Authors:** Kai Shi, Qiaohui Liu, Qingrong Ji, Qisheng He, Xing-Ming Zhao

**Affiliations:** College of Computer Science and Engineering, Guilin University of Technology, Guilin, Gaungxi 541004, China; Guangxi Key Laboratory of Embedded Technology and Intelligent Systems, Guilin University of Technology, Guilin, Gaungxi 541004, China; College of Computer Science and Engineering, Guilin University of Technology, Guilin, Gaungxi 541004, China; College of Computer Science and Engineering, Guilin University of Technology, Guilin, Gaungxi 541004, China; College of Computer Science and Engineering, Guilin University of Technology, Guilin, Gaungxi 541004, China; Huzhou Central Hospital, Affiliated Central Hospital Huzhou University, Huzhou, Zhejiang 313000, China; Institute of Science and Technology for Brain-Inspired Intelligence, Fudan University, Shanghai 200433, China

**Keywords:** disease prediction, gut microbiome, machine learning, metagenomics, phylogenetic tree

## Abstract

The gut microbiota plays a vital role in human health, and significant effort has been made to predict human phenotypes, especially diseases, with the microbiota as a promising indicator or predictor with machine learning (ML) methods. However, the accuracy is impacted by a lot of factors when predicting host phenotypes with the metagenomic data, e.g. small sample size, class imbalance, high-dimensional features, etc. To address these challenges, we propose MicroHDF, an interpretable deep learning framework to predict host phenotypes, where a cascade layers of deep forest units is designed for handling sample class imbalance and high dimensional features. The experimental results show that the performance of MicroHDF is competitive with that of existing state-of-the-art methods on 13 publicly available datasets of six different diseases. In particular, it performs best with the area under the receiver operating characteristic curve of 0.9182 ± 0.0098 and 0.9469 ± 0.0076 for inflammatory bowel disease (IBD) and liver cirrhosis, respectively. Our MicroHDF also shows better performance and robustness in cross-study validation. Furthermore, MicroHDF is applied to two high-risk diseases, IBD and autism spectrum disorder, as case studies to identify potential biomarkers. In conclusion, our method provides an effective and reliable prediction of the host phenotype and discovers informative features with biological insights.

## Introduction

The human microbiota, which comprises bacteria, archaea, protists, fungi, and viruses, inhabits the human body and forms a complex and diverse ecosystem. Mounting evidence indicates that dysregulation of the human microbiome is associated with numerous diseases including obesity, diabetes, inflammatory bowel disease (IBD), cardiovascular diseases (CVD), and autism spectrum disorders (ASD), among others [1]. Patients with CVD exhibit abnormal gut microorganisms, where the abundance of certain gut microbiota (such as *Prevotella*, *Bifidobacterium*, and *Firmicutes*) in hypertensive patients is significantly lower than that in healthy individuals [2]. In addition, researches have revealed that disruptions in the intestinal microbiome can influence the progression of ASD in children, owing to the intricate relationship between the nervous system and gastrointestinal tract [3]. For example, the production of butyrate by *Bacteroides* and *Prevotella* has been found to potentially promotes carbohydrate depletion in individuals with ASD [4]. Therefore, investigating disease mechanisms from a microbiome perspective may offer new insights into disease diagnosis, treatment, and prognosis.

With advances in sequencing technology, microbial data have been generated and stored in public repositories (MGnify [5], GMrepo v2 [6], Qiita [7], and the National Centre for Biotechnology Information [8] *et al*.), making it possible to explore diseases in a data-driven manner. Currently, machine learning (ML)-based approaches are widely applied to various microbiome analysis tasks, including phenotype prediction, microbial feature classification, studying microbiome component interactions, and monitoring changes in microbiome composition [9, 10]. In this study, we focused on predicting the host phenotype using metagenomic data.

The human microbiome contains rich data expressed in different feature forms such as operational taxonomic units, amplicon sequence variants, and k-mer representation. Usually, classical ML methods, such as SVM and random forest (RF), make host phenotypic predictions based on these features or newly generated feature representations [11–13]. In addition, several studies have focused on large-scale meta-analyses to validate findings across cohorts using 16S rRNA, shotgun metagenomic, and other omics data [12, 14, 15]. In exploring colorectal cancer, Wirvel *et al*. [12] revealed disease-specific microbial signatures in a meta-analysis, and Thomas *et al*.[14]identified replicable cross-cohort microbial signatures. Jiang *et al*. [15] attempted to identify consistent microbial alterations in common intestinal diseases through meta-analysis. Regarding the input feature problem, Giliberti *et al*. [16] performed multiple case–control studies and indicated that the presence, rather than the relative abundance, of microbial taxa is important for classification models. To evaluate the model for host phenotype classification, Pasolli *et al*. [17] proposed a comprehensive meta-analysis framework that applied cross-validation, cross-study validation, and cross-disease validation to ML methods. Li *et al*. [18] following this evaluation principle conducted a comprehensive meta-analysis of 20 diseases in 83 cohorts and demonstrated that different diseases, sequencing types, and sample sizes were important factors for ML classifier performance. Moreover, ML-based web services and toolboxes have been developed to facilitate phenotype classification tasks, such as ML Repo [19], SIAMCAT [20], GutBalance [21], MarkerML [22], and DisBalance [23] *et al*. On the other hand, it is well-known that different taxonomic ranks can significantly impact the learning and predictive performance of ML models, which poses challenges in developing robust ML models.

Recently, deep learning approaches have become popular solutions for host phenotype classification in case–control microbiome studies [10]. The basic concept is to transform microbiota abundance data into a low-dimensional representation for downstream analysis, where relevant works refer to the method using multilayer perceptron neural networks (MLP) with augmented samples [24], ensemble CNN method considering the inherent correlation between taxonomy [25], and methods obtaining feature representation via autoencoder [26] or deep variational information bottlenecks [27]. With the popularity of graph neural network, one relevant work is that ensemble GraphSAGE models were used to construct a disease network module for metagenomic disease prediction from an OUT table [28]. Moreover, Liao *et al*. [29] constructed an inter-host microbiome similarity graph using the latent features of samples learned from a deep adaptation network, and the GCN mode was trained to classify disease status. Although these methods achieved satisfactory results, they neglected the relationships among taxonomic hierarchical structures. Other researchers integrated the phylogenetic tree to improve prediction performance, where the phylogenetic tree is traversed according to different strategies for visiting tree nodes (position mapping and tree traversal). Reiman *et al*. [30] initially applied the CNN method to predict host phenotypes by embedding microbial phylogenetic trees and the abundance of microbial taxa in a 2D matrix, maintaining spatial and quantitative information from metagenomic data. Li *et al*. [31] further extended phylogenetic information integration with graph normalization by considering the children of nodes, heights of layers, and patristic distance. Moreover, Chen *et al*. [32] proposed a framework to obtain an embedded taxonomic representation by level and post-order traversals of the phylogenetic tree.

Although significant advancements have been made in the field of phenotypic prediction, notable limitations exist despite the extensive efforts of previous researchers. Traditional ML approaches in meta-analysis are capable of yielding profound discoveries, but they fall short in terms of accuracy and cross-cohort predictions. In contrast, deep learning methods exhibit high predictive accuracy but operate as black boxes, lacking model interpretability. Moreover, these methods overlook the prevalent issues of imbalanced sample classes within a dataset, which widely exists in clinical scenario. Minority classes may be underrepresented when machine-learning methods are trained on class-imbalanced data, resulting in poor predictive performance [33]. In recent years, the deep forest (DF) tree model [34] has been acknowledged for its interpretability and has been applied in metagenomic analysis [35], but still fail to overcome these limitations [36].

Inspired by previous work [32, 34, 37], we address the challenges of sample class imbalance and high-dimensional feature by introducing a new framework, named Microbiome Hybrid Deep Forest (MicroHDF), to enhance the accuracy of host phenotype prediction based on metagenomic data. We first design and integrate two types of forest units into an ensemble framework with cascading layers. One unit employs a RF combined with affinity propagation (AP) clustering and stratified under-sampling(RF-CUS [37]), specifically to address class imbalance. The other unit uses an extreme tree forest (ERTs [38]) based on data complexity reduction to handle the challenges of high-dimensional data. The phylogenetic tree, which depicts evolutionary history and relationships among species, is used to generate a novel feature matrix incorporating both the structural information of the tree and species abundance data. This new feature matrix, along with the species abundance matrix, are then introduced into the ensemble framework through two distinct channels. Moreover, robust features for the classification task are generated by aggregating features from these two channels. The experimental results show that MicroHDF outperform existing state-of-the-art methods on 13 publicly available datasets of six different diseases. Our method provides an effective and reliable prediction of the host phenotype and discovers informative features with biological insights.

## Materials and methods

**Figure 1:**
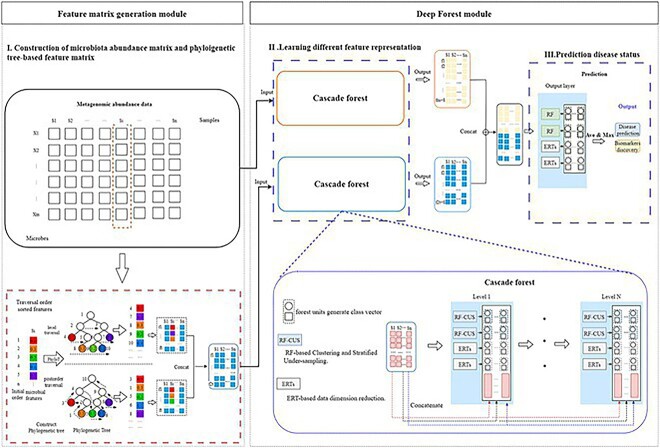
Overall workflow of MicroHDF. Metagenomic abundance data embedded into the phylogenetic tree is transformed to new feature matrixes by two different tree traversals in the feature matrix generation module. Next, different feature representation are learned from the cascade layers with different DF-based units. Finally, the learned features are aggregated to output layer and set to perform classification tasks.

MicroHDF is composed of two modules: generating feature matrix and DF module. An overview of MicroHDF is shown in Fig. 1, in which the microbial abundance profile and phylogenetic tree-based feature matrix are the inputs of the modified DF module. New feature representations are learned on two channels, considering different data information from the DF units. Finally, class distributions are used to make decisions.

### Construction of phylogenetic tree-based feature matrix

Numerous recent studies have highlighted the potential improvement of model by incorporating phylogenetic information, given that phylogenetic analysis considers the evolutionary relationships among species [30–32, 39]. Typically, phylogenetic relationships are illustrated in the form of a phylogenetic tree, in which related organisms tend to exhibit similar characteristics. In this study, we initially utilize PhyIoT [40] to construct a least-prunes phylogenetic tree based on the taxonomic annotations of microbial species. Furthermore, we integrate the phylogenetic tree with microbial abundance profiles.

In the light of reported research, the taxonomic spatial information can be gleaned through traversing the phylogenetic tree [32]. In this study, we investigate two order templates employed in popular traversal algorithms for tree structure post-order and level traversal. With the post-order traversal-based order template, taxa are rearranged in a left-to-right sequence, where groups of taxa sharing the same ancestry reflect the relative temporal order of species nodes during the evolutionary process. Conversely, the order template based on level traversal captures every node at each level of the tree, from top to bottom and left to right, associating tree levels with diverse evolutionary phases and genetic information and representing the node evolutionary stage. Subsequently, the abundance of taxa in a sample is used to populate the order templates as new feature vectors. Finally, two phylogenetic tree-based feature matrices are constructed based on the new feature vectors (details are provided in Fig. S1).

### Random forest units for dealing with metagenomic data

It is widely acknowledged that analysing metagenomic data is challenging because of imbalanced samples and high-dimensional features. Traditional machine-learning approaches typically address sample imbalances or focus on overcoming the challenges associated with high-dimensional features, whereas only a few models consider both aspects simultaneously. Inspired by [37], we devised various RF-based units (RF-CUS and ERTs)to address this challenge. The standard operational process of the RF primarily involves bootstrap sampling and feature selection. The advantages of the RF are its efficient training process and reduced susceptibility to overfitting.

In this work, we apply a DF-based unit (RF-CUS) [37] to deal with the imbalanced data, which leverage a class-rebalancing strategy and RF ensemble prediction (Fig. S2). The training set undergoes AP clustering to cluster majority class samples. The AP clustering algorithm can dynamically discover cluster centers and autonomously assign data points to clusters. In our study, we employed the Bray–Curtis dissimilarity metric for sample similarity calculations. When the initial method results in too few or too many clusters that fail to represent the underlying data structure accurately, we adjust the number of iterations and convergence to obtain the optimal number of clusters (typically *k* = 15) under the best classification performance. Furthermore, a fallback mechanism is designed to replace AP algorithm with K-means clustering if the model fails to converge. Subsequently, stratified undersampling is performed within different clusters to generate subsets. Each cluster is sampled at a specific ratio according to the size of the small sample dataset, and this process is repeated N times. This results in the creation of a serial of sample sets for majority classes. Bootstrap sampling is repeatedly applied to the minority class samples to generate an equal number of sample sets. Subsequently, pairwise sampling is conducted to create class-balanced training sets from these two sequences of sample subsets. Finally, the basic RF classifier is employed on each of the balanced training sets, and the classification results are aggregated by voting to formulate the final prediction. Stratified undersampling performed through AP clustering ensures the preservation of valuable information from the majority class, while achieving a balanced merging of sampled datasets. Additionally, the RF ensemble framework effectively rebalances the training dataset to enhance the prediction accuracy.

Moreover, dimensionality reduction is an effective approach for managing the high-dimensional features of microbiome data. To enhance the prediction performance, we adopt ERT-based units to reduce the feature dimension. Initially, the Linear discriminant analysis (LDA) scores are utilized to rank the differential taxa when distinguishing disease and control samples [41]. Subsequently, the feature subset with the most discriminative ability is identified from the ranked feature dataset using a wrapper method, where ERTs served as the basic classifier, and sequential forward selection is adopted as the search strategy. Finally, the local optimal feature subset is employed to train the ERTs, utilizing these features as candidates to be randomly selected to obtain the optimal split node.

### Architectures of the MicroHDF

Our framework follows a three-step procedure: (i) construction of the microbial abundance matrix and phylogenetic tree-based feature matrix to facilitate proper learning; (ii) utilization of DF units to learn different feature representations; and (iii) utilization of class distributions to make predictions. As depicted in Fig. 1, the microbiota abundance and phylogenetic tree-based features are incorporated into the two cascade forest structures within the DF module. Taking cues from deep neural networks, the cascade forest structure resembles a multilayered approach, where feature information progresses from one level to the next. Specifically, the RF-CUS and ERTs serve as embedded units to address sample imbalances and high-dimensional feature issues at each level. Essentially, each level functions as an ensemble of decision-tree forests. Subsequently, the class vectors representing the estimated class distribution for a given sample are concatenated with the raw feature vector and inputted to the subsequent cascade level. Following multilayer operations, new feature representations in the form of estimated class distributions are obtained. Furthermore, new feature representations derived from the abundance feature matrix are combined with those derived from the phylogenetic tree and inputted into the final layer. Here, the RF and ERTs serve as the basic units for the final layer, and the class distribution vectors from different units are averaged to yield the final prediction. This systematic process is formalized as follows:


(1) \begin{equation*} {h}_{l+1}={f}_{l+1}\left({h}_l\right)\Big\Vert x \end{equation*}

(2) \begin{equation*} {f}_{l+1}\left({h}_l\right)=\underset{j}{\Big\Vert}\left(\underset{k=1}{\overset{n}{\Big\Vert }}{\left[{p}_{1,0},{p}_{2,1}\right]}_k^j\right) \end{equation*}

where *x* denotes the input sample vector, ${h}_l$ denotes a new feature representation after *l-th* cascade layer, *f* is the ensemble tree model, and || denotes the vector concatenation operation. In Formula 2, for the cascade layer *l-th*, tree model *j* is RF-CUS and ERTs, and on the last layer *j* is RF and ERTs. *k* is the modal unit index. Moreover, $\left[{p}_{1,0},{p}_{2,1}\right]$is the class probability vector of the mode unit used for binary classification.

### Batch effect adjustment

In cross-study validation, we corrected batch effects between datasets using the R package MMUPHin, where the variable ‘dataset’ and sample labels in the metadata are as covariates. PERMANOVA test was conducted to evaluate the effect of batch adjustment, which can be performed with the adonis function in the vegan package in R.

### Performance evaluation

MicroHDF is a deep learning approach based on a DF framework, wherein each cascade layer is composed of ensemble RFs. Consequently, the ensemble of decision-tree forests provides interpretability, and the significance of features in classification tasks can be quantified using pertinent indicators. Typically, classical assessment metrics are used in decision trees, such as information gain or Gini impurities. The feature importance value (FIV) for a specific feature can be viewed as the ratio of its information gain values relative to those of all features in the trees of the cascade structure.


(3) \begin{equation*} {F}_l^d={\sum}_{n=1}^N{\sum}_{t=1}^T{l}_{n,t}^{d,l} \end{equation*}

(4) \begin{equation*} {F}_d=\frac{\sum_{l=1}^L{F}_l^d}{\sum_{d=1}^D{\sum}_{l=1}^L{F}_l^d} \end{equation*}

where in the *l-th* layer, ${F}_l^d$ denotes the sum of the information gain of the feature *d-th* in the *t* tree of the *n* forest. Equation (4) expresses the normalized FIV for feature *d*.

To demonstrate the effectiveness of MicroHDF, we utilize five-fold cross-validation repeated ten-fold, to assess the performance of the model. The evaluation is based on the area under the receiver operating characteristic curve (AUC) and the area under the precision-recall curve (AUPR), with the AUPR being particularly informative in the assessment of imbalanced data [42]. Furthermore, in addition to the aforementioned metrics, we employ other quantitative measurements, including accuracy, recall, and F1-score.

## Results

### Datasets and settings

To benchmark with other methods, we collect species-relative abundance profiles of the human gut microbiota from 13 distinct publicly available datasets covering six different diseases (Table 1): obesity [43] (Obesity), colorectal cancer [44] (Colorectal), liver cirrhosis [45] (Cirrhosis), type 2 diabetes in China [46] (C-T2D), type 2 diabetes in Europe [47] (EW-T2D), inflammatory bowel disease(IBD [48], NielsenHB_2014 [49], IjazUz_2017 [50], ICDf [51]) and ASD (Li_ASD [52], Chen_ASD [53], Arizo_ASD [54], Dan_ASD [55]). These datasets were selected as benchmarks to evaluate model performance across different sample class imbalance ratio (IR). Additionally, the IBD and ASD datasets were used for further cross-study validation. The IR is defined as the ratio of the majority class to the minority class, which conveys the degree of sample-type imbalance. To facilitate cross-validation and cross-study validation, we calculated the relative abundances of taxa and removed batch effects by using the R package MMUPHin. For IBD disease, the ‘dataset’ variable can explain 34.49% of the variance in microbial profiles between studies (IjazUz_2017, ICDf), which decreased to 23.52% after batch effect adjustment. For ASD disease (Li_ASD, Dan_ASD), the variance explained by the ‘dataset’ variable is 20.01% before and 18.74% after batch effect adjustment.

**Table 1:** The detailed information of 13 cohorts used in our study. The first six cohorts are used for the five-fold-cross-validation experiment. The inflammatory bowel disease and ASD disease cohorts are used for cross study experiment.

Disease name	Cohort name	Disease cases	Healthy controls	Total number samples (TN)	Feature numbers	Imbalance Ratio (IR)	Reference
obesity	Obesity	164	89	253	465	1.84	[17, 43]
colorectal cancer	Colorectal	48	73	121	507	1.52	[17, 44]
liver cirrhosis	Cirrhosis	118	114	232	541	1.03	[17, 45]
Type 2 diabetes	EW-T2D	53	43	96	381	1.23	[17, 47]
	C-T2D	170	174	344	606	1.02	[17, 46]
Inflammatory bowel disease	IBD	25	85	110	443	3.4	[17, 48]
	NielsenHB_2014	148	248	396	292	1.67	[16, 49]
	IjazUz_2017	56	38	94	230	1.34	[16, 50]
	ICDf	44	38	82	246	1.15	[30, 51]
Autism spectrum disorders	Li_ASD	120	401	521	1342	3.34	[52]
	Chen_ASD	76	47	123	455	1.61	[53]
	Arizo_ASD	20	20	40	841	1.0	[54]
	Dan_ASD	143	143	286	1120	1.0	[55]

Within the cascade layers of MicroHDF, the number of decision trees is an essential hyperparameter and it was set to 100. Additionally, the undersampling ratio in the RF-CUS unit was set to 0.4. At the same time, we adopt RF-CUS*2 and ERT*2 as the forest units, and more detailed information is in Supplementary (Table S1).

### Simulation Study

**Figure 2:**
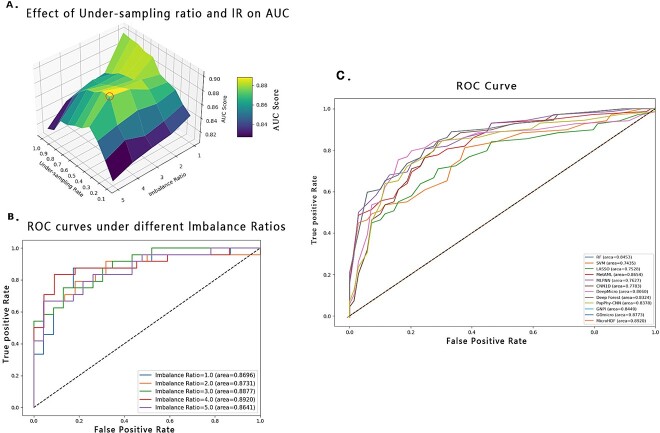
The impact of performance under different class IRs and under-sampling ratios on MicroHDF in the synthetic dataset.

We initially apply MicroHDF to synthetic datasets to analyse the impact of different imbalance and under-sampling ratios for RF-CUS units. To simulate species abundance, we employ SparseDOSSA2 (R package) to generate synthetic microbiome data [56]. Figure 2A illustrates the prediction performance of the MicroHDFs on synthetic datasets with different IR and under-sampling ratio. Furthermore, on the simulated dataset, the method with under-sampling ratio of 0.4 and IR of four yielded the top performance with 0.892 AUC, as depicted in Fig. 2B. Under these conditions, our method exhibits superior performance compared to the other methods on synthetic metagenomic data, as depicted in Fig. 2C.

### Comparisons with state-of-the-art methods on real datasets

In this section, we benchmark our model to state-of-the-art methods for host phenotype prediction, including three ensemble learning methods, MetAML [17], RF and GMHI [57], two classical ML methods, SVM and LASSO, and seven deep-learning-based state-of-the-art models: MLPNN [58], CNN1D [59], DeepMicro [26], Deep Forest [60], PopPhy-CNN [30], GNIP [31], and GDmicro [29].

**Figure 3:**
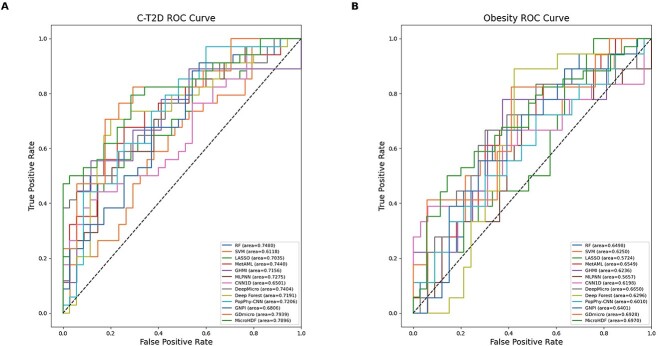
ROC-AUC comparison between MicroHDF and the other 12 models. (A) the C-T2D disease dataset with low IR. (B) the obesity disease dataset with high IR.

To ensure a fair comparison, all experiments are conducted on different disease datasets with different class IRs, whereas five-fold cross-validation is performed for ten repetitions. Figure 3(A) and (B) present the ROC-AUC in the C-T2D (IR:1.02, TN:344) and obese (IR:1.84, TN:253) cohorts, respectively. In the Obesity cohort, MicroHDF achieves 0.6970 ± 0.0125 AUC and 0.7518 ± 0.0124 AUPR outperforming all the other methods. In the C-T2D cohort, although MicroHDF obtains a competitive AUC of 0.7896 ± 0.0049, less than the first-ranked method (GDmicro), our method has an AUPR of 0.7592 ± 0.0147 (Table S3), which is a 1.8% improvement over GDmicro (0.7412 ± 0.0350). To achieve a comprehensive assessment, we further investigate different metrics such as AUC, AUPR, accuracy, recall, and F1-score, on Cirrhosis (IR:1.03, TN:232) and IBD (IR:3.4, TN:110) cohorts. The result presents that the performance of MicroHDF is superior to that of the other methods not only in low-IR cohort(Cirrhosis AUC: 0.9469 ± 0.0076, AUPR: 0.9480 ± 0.0049, F1-score: 0.8891 ± 0.0101), but also in higher IR cohort(IBD AUC: 0.9182 ± 0.0098, AUPR: 0.7962 ± 0.0117, F1-score: 0.8959 ± 0.0134), despite some methods yield a slightly higher accuracy and recall (GDmicro) compared to our method (Table 2). For the EW-T2D and Colorectal datasets, the MicroHDF also achieves notable performance improvements (Table S3). In addition, the Friedman test was used to analyse the performance difference among the 13 methods on six datasets. The Friedman statistical values and the corresponding p values for AUC and AUPR are 50.26 (*P* = 1.26e-06), and 45.27 (*P* = 9.25e-06), respectively (N = 6, K = 13), as shown in supplement Tables S5 and S6. The result indicates that our method exhibits significant differences compared to the other 12 algorithms. Our results indicate that MicroHDF consistently demonstrates competitive performance, achieving higher evaluation metric results across different class IRs. In contrast, ensemble method-based approaches generally exhibit better prediction performance than single-learner paradigms (e.g. SVM and LASSO) for microbial abundance data, possibly because of their capacity to mitigate overfitting risks. Furthermore, although GNPI and PopPhy-CNN demonstrated slightly inferior performance compared to MicroHDF, their excellence may be attributed to the incorporation of phylogenetic tree information. In addition, although the transfer-learning-based method (GDmicro) may outperform MicroHDF on certain metrics, this can be attributed to the use of more samples to learn from different domains using semi-supervised learning and domain adaptation.

**Table 2:** The performance comparison between MicroHDF and 12 baseline methods on cirrhosis and IBD disease datasets, with the best result highlighted in bold and the second rank result underlined.

Dataset	Methods	AUC(%)	AUPR(%)	Accuracy(%)	Recall(%)	F1-score(%)
Cirrhosis IR = 1.03	RF	93.35	93.28	88.35	88.27	87.23
	SVM	93.20	92.28	83.19	85.79	85.59
	LASSO	88.95	88.80	77.59	79.00	78.79
	MetAML	92.19	93.55	87.70	87.40	87.89
	GHMI	90.51	89.01	81.91	86.22	81.76
	MLPNN	92.14	92.13	84.06	84.51	84.23
	CNN1D	90.57	90.48	80.56	82.84	82.68
	DeepMicro	88.50	93.20	81.70	74.00	80.00
	Deep Forest	92.03	91.13	86.24	86.24	81.23
	PopPhy-CNN	90.53	91.40	84.32	83.32	80.76
	GNPI	92.23	93.87	89.36	87.23	81.07
	GDmicro	94.63	93.82	91.13	92.32	88.39
	MicroHDF	94.69	94.80	89.93	91.26	88.91
IBD IR = 3.4	RF	87.36	75.21	82.70	80.90	74.87
	SVM	75.97	61.66	80.00	78.13	72.51
	LASSO	76.52	58.96	78.18	76.36	68.62
	MetAML	88.20	71.55	75.90	76.00	75.70
	GHMI	85.74	76.28	80.91	83.72	87.02
	MLPNN	77.83	72.40	82.72	68.18	68.72
	CNN1D	84.64	66.76	81.81	75.45	74.73
	DeepMicro	85.00	74.20	77.27	80.00	85.33
	Deep Forest	84.30	76.35	78.18	78.16	73.69
	PopPhy-CNN	83.50	70.00	72.18	78.18	77.39
	GNPI	87.53	73.12	79.94	79.94	72.84
	GDmicro	88.58	78.89	87.27	87.24	87.83
	MicroHDF	91.82	79.62	86.35	86.49	89.59

On the other hand, several studies have reported that models applied to obesity cohorts often exhibit lower performance. Consistent with these findings, our results also indicate only a slight improvement in performance. It is possible that obesity is a complex disease, caused by the interaction of genetic, environmental, and lifestyle factors. Microbial disorders may be just one of the factors contributing to the disease.

### Cross-study validation

The cross-validation discussed in the previous section allows the evaluation of disease phenotype predictability in a single cohort. However, it may not effectively assess the generalizability of the model to independent validation samples, which is a scenario that is more relevant to a clinical setting. We addressed this question by applying a cross-study strategy to validate the performance of the model.

**Figure 4:**
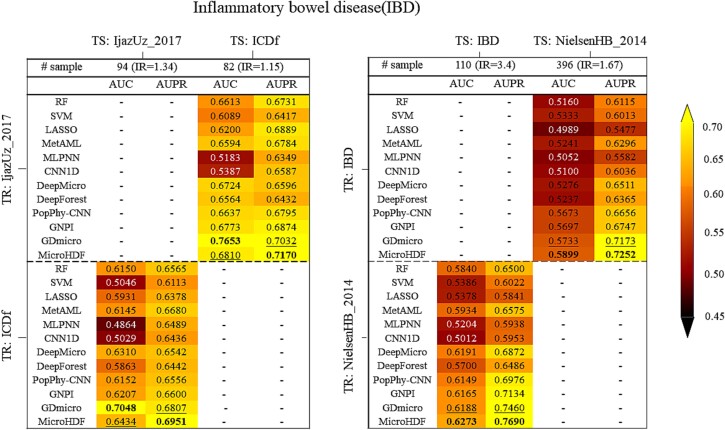
AUC and AUPR through cross-study analysis for IBD disease. The model was generated through training (TR) and then applied to test (TS). In bold we report the top value for each setting.

**Figure 5:**
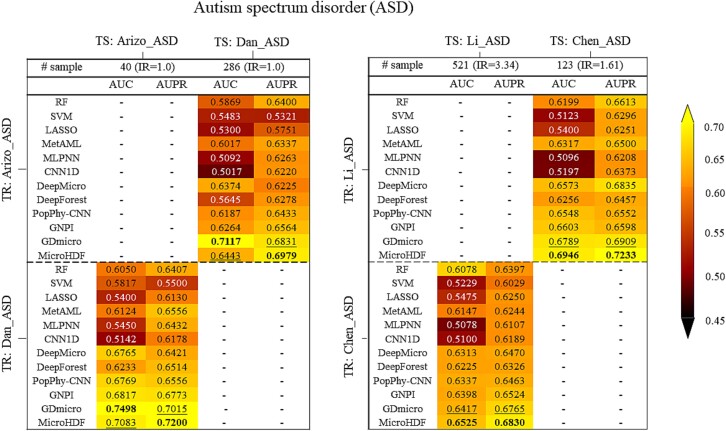
AUC and AUPR through cross-study analysis for ASD disease. The model was generated through training (TR) and then applied to test (TS). In bold we report the top value for each setting.

First, we focus on IBD, referring to two types of datasets with differential IR (i.e. slightly different IR between the two cohorts and a substantial difference in IR between the two cohorts) across four different cohorts: IjazUz_2017(IR:1.34), ICDf(IR:1.15), IBD(IR:3.4), and NielsenHB_2014(IR:1.67). The generalization of the model is assessed by training the model on the cohort (TR) and applying it to a test cohort (TS). Figure 4 shows the cross-study validation results for the datasets with different IR values. Our method achieves competitive performance compared to baseline methods. Specifically, the AUC value of MicroHDF in the IBD cohort increases slightly from 0.5899 to 0.6273 when generated from the NielsenHB_2014 cohort. In addition, MicroHDF outperforms all the other methods in terms of AUPR, which increased by ~ 2% for the second-rank method (GDmicro). A similar analysis is performed on ASD in four distinct cohorts (Arizo_ASD(IR:1), Dan_ASD(IR:1), Li_ASD(IR:3.34), and Chen_ASD(IR:1.61)), each representing different population characteristics from various countries. Despite clear cohort effects, we observe generalizations from one study to another, as shown in (Fig. 5). Validation of the Arizo_ASD and Dan_ASD cohorts reveals that the AUC value for MicroHDF is lower than that for GDmicro when constructed on Dan_ASD and Arizo_ASD, respectively. However, for the validation of the datasets (Li_ASD and Chen_ASD) with a higher IR difference, our method has an AUC of 0.6946 and 0.6525, which is higher than that of the top tools. Notably, our method achieves the top AUPR compared to other methods for cross-study validation. Additionally, we conducted performance comparisons across multiple cohorts for the same disease, with the results shown in Fig. S3. In conclusion, the experimental results indicate that a larger number of samples in the training dataset corresponds to improved model performance, and training on a dataset with a high-class IR may result in reduced generalization.

## Components affecting prediction performance

### Effect of phylogenetic information

To evaluate the effectiveness of the model, we conduct additional experiments on seven cohorts to compare prediction performance with and without phylogenetic tree information. Table 3 presents the results of the study, in which the feature format based on phylogenetic trees include raw features (L), raw features (P), and raw features (L + P), representing information obtained through level traversal, post-order traversal, and a combination of both. The raw feature (O) indicates that the model trained the dataset without phylogenetic tree information. Across several cohorts, spatial information from phylogenetic trees can improve the final prediction accuracy by 1%–5%, although there is variation in performance among different profiles with distinct taxonomic tree spatial information. Detailed model predictions are provided in Table S7.

**Table 3:** Comparison with and without phylogenetic tree information and different microbial features representation.

Dataset	Raw feature(O) AUC(%)	Raw feature (O + L) AUC(%)	Raw feature (O + P) AUC(%)	Raw feature (O + L + P) AUC(%)
IBD	89.88	91.29	91.29	91.82
Obesity	68.09	68.92	69.06	69.70
Colorectal	68.90	70.62	72.01	76.64
EW-T2D	68.09	68.92	69.76	73.18
Cirrhosis	94.48	94.49	94.52	94.69
C-T2D	77.86	77.96	78.42	78.96
Li_ASD	76.48	78.48	78.96	80.66

Furthermore, considering that different taxonomic levels of microorganisms can influence model performance, we conducted experiments to compare the performance of various models using an integrated abundance data matrix across all hierarchical levels (Table S8) as well as different taxonomic ranks (Table S12). In six datasets, our method, MicroHDF-S, which does not incorporate phylogenetic tree information, achieved competitive results, attaining the second highest AUC of 94.41 and 78.33 for the Cirrhosis and C-T2D datasets, respectively. The most models show performance improvements due to the inclusion of additional features and implicit hierarchical information. Furthermore, we compared our method, MicroHDF-T, which integrates species-level abundance profiles with phylogenetic tree information. This approach yielded the highest AUC of 94.69 and AUR of 94.8 for the Cirrhosis dataset, as well as the second highest AUC of 69.70 and AUR of 75.18 for the Obesity dataset. It also demonstrated competitive performance across other datasets, outperforming most models that rely solely on aggregated abundance data across all hierarchical levels. Therefore, for phenotypic classification tasks, the effective incorporation of phylogenetic tree information can enhance model performance.

### Effect of different architectures

In addition, we compare the performance of a single-channel module with that of a two-channel module based on microbial abundance and phylogenetic tree features. As shown in Table S9, the two-channel module yields improved prediction performance compared to the single-channel module across seven metagenomic datasets. The average AUCs and F1 scores increased by 1%–3% across the different datasets. The single-channel learning module utilizes a modified DF model to simultaneously learn the microbial abundance profile and phylogenetic tree features. The two-channel learning module consists of two separate and identical modules, each addressing the sample class imbalance and high dimensionality (Fig. S4). It enables independent learning of the embeddings of phylogenetic tree features and microbiological abundance features, with the learned embeddings from both channels combined as inputs to the prediction module.

### Experimental ablation study

To further evaluate the capabilities of MicroHDFs in managing imbalanced class distributions and high-dimensional microbiota data, we conduct an ablation study to compare the classification performance of RF-CUS and ERTs as embedded units in cascade layer settings. For this purpose, we derive the following variants of our model to assess the impact of each unit:

gcForest: Two fully randomized forests and two RFs embedded in cascade layers.

The MicroHDF-CUS uses only four RF-CUS units embedded in the cascade layers of the DF.

employing only four ERTs units embed in the cascade layers of the DF.

**Figure 6:**
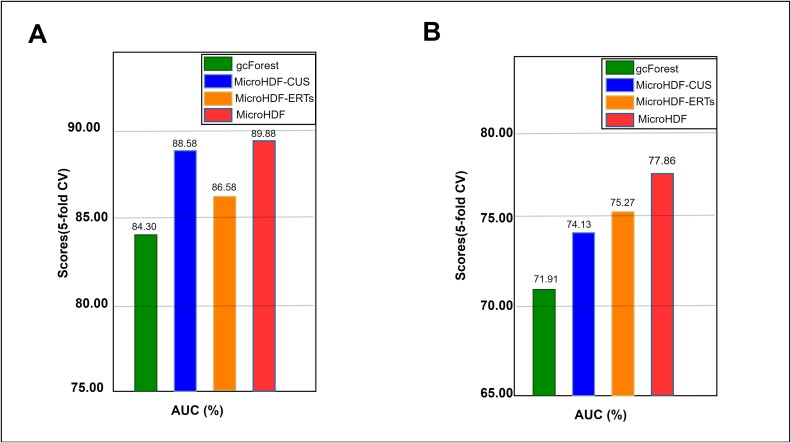
Comparative analysis between MicroHDF and its variants on IBD cohort (A) and C-T2D cohort (B).

Figure 6 illustrates that the MicroHDF consistently outperforms the other variants. The RF-CUS unit plays a crucial role in addressing imbalanced sample distributions, as MicroHDF-CUS achieves comparable AUC performance on the IBD dataset. Moreover, MicroHDF-CUS demonstrates a higher performance than MicroHDF-ERTs and gcForest, highlighting the contribution of the RF-CUS unit in enhancing the prediction accuracy of our model. Additionally, the ERTs unit enhances the prediction capability of MicroHDF, as evidenced by the higher AUC values of MicroHDF-ERTs compared with MicroHDF-CUS on the C-T2D dataset, which have balanced sample distributions.

### Interpretable analysis of important features

MicroHDF addresses the issue of limited model interpretability in most deep-learning methods by quantifying the contribution of microbes and detecting the most discriminative features relevant to the predicted class. By sorting the features through FIV, we are able to identify the most important bacteria for each dataset with a predominant influence on disease predictions. In Tables 4 and 5, we present the top 20 microbes based on their FIV for two common diseases: IBD and ASD, the top 50 microbes related to the disease are detailed in Tables S10 and S11. Additionally, we use linear discriminant analysis Effect Size (LEfSe) to analyse the relationship between microbiota and disease. Linear Discriminant Analysis (LDA) scores are used to quantify the extent of differences, with larger values indicating more significant differences.

**Table 4:** Prediction results of the top 20 IBD-associated microbes.

Disease	Rank	FIV	LDA score	Microbe	Evidence
IBD	1	0.2818	3.252	*Unclassified Oscillibacter*	PMID:35533243
	2	0.2364	3.427	*Bacteroides intestinalis*	PMID:25307765
	3	0.1745	3.165	*Odoribacter splanchnicus*	PMID:33281770
	4	0.1545	3.244	*Ruminococcus lactaris*	PMID:33330572
	5	0.1364	2.990	*Roseburia hominis*	PMID:33135936
	6	0.1091	3.210	*Bifidobacterium bifidum*	PMID:29796620
	7	0.1091	3.201	*Alistipes finegoldii*	PMID:26288277
	8	0.1090	3.171	*Ruminococcus bromii*	PMID:31378787
	9	0.1000	2.777	*Lachnospiracea bacterium 8_1_57FAA*	PMID:30936547
	10	0.0818	2.689	*Bacteroides vulgatus*	PMID:32761124
	11	0.0808	2.620	*Lachnospiracea bacterium 8_1_58FAA*	PMID:30936547
	12	0.0799	2.611	*Akkermansia muciniphila*	PMID:32761124
	13	0.0784	2.602	*Faecalibacterium prausnitzii*	PMID:29796220
	14	0.0784	2.597	*Alistipes shahii*	PMID:26288277
	15	0.0771	2.597	*Bacteroides cellulosilyticus*	PMID:30124831
	16	0.0769	2.577	*Coprococcus comes*	PMID:24629344
	17	0.0768	2.564	*Odoribacter_unclassified*	PMID:26789999
	18	0.0767	2.537	*Butyrivibrio_crossotus*	PMID:32761142
	19	0.0757	2.476	*Coprococcus_sp_ART55_1*	Unconfirmed
	20	0.0756	2.426	*Barnesiella_intestinihominis*	Unconfirmed

**Table 5:** Prediction results of the top 20 ASD-associated microbes.

Disease	Rank	FIV	LDA score	Microbe	Evidence
ASD	1	0.0576	4.524	*Eubacterium limosum*	PMID:32867322
	2	0.0575	4.503	*Ruminococcaceae UCG-003*	PMID:32546239
	3	0.0563	4.498	*Clostridium sensu stricto 13*	Unconfirmed
	4	0.0533	4.441	*Prevotella*	PMID:28122648
	5	0.0533	4.441	*Lachnospiraceae_NK4A136*	PMID:34562157
	6	0.0532	4.389	*Lachnospira*	PMID:35275534
	7	0.0531	4.328	*Clostridium perfringens*	PMID:32312186
	8	0.0530	4.328	*Bifdobacterium*	PMID:32867322
	9	0.0512	4.220	*Sulfurovum*	Unconfirmed
	10	0.0503	4.216	*Pedobacter*	Unconfirmed
	11	0.0500	4.206	*Bacteroides_uniformis*	PMID:20613793
	12	0.0497	4.191	*Ruminococcus_bicirculans.1*	PMID:30567928
	13	0.0496	4.179	*Blautia glucerasea*	PMID:28429209
	14	0.0496	4.143	*Alistipes_putredinis.1*	PMID:30567928
	15	0.0496	4.124	*Bacteroides_stercoris*	PMID:22180058
	16	0.0486	4.024	*Bacteroides_uniformis.2*	PMID:26789999
	17	0.0485	4.003	*Bacteroides_vulgatus8*	PMID:22202440
	18	0.0485	4.002	*Alistipes_putredinis.2*	PMID:24130822
	19	0.0482	4.010	*Bacteroides_vulgatus11*	PMID:20603222
	20	0.0479	4.122	*Akkermansia muciniphila*	PMID:24130822

IBD is a general term encompassing ulcerative colitis (UC) and Crohn’s disease (CD). Recently, microorganisms are shown to have a significant impact on the development, progression, and treatment of IBD [61]. Based on our findings, *Unclassified Oscillibacter* is the most associated with IBD which is the top of the FIV. *Odoribacter splanchnicus* ranks third, is generally discovered to decrease in abundance, and is linked to the early stages of IBD [62]. *Alistipes finegoldii* ranks seventh, and has been identified as a microbial driver of IBD [63–65]. Our model-computed FIV is consistent with the trend in the results of the LEfSe analysis. In other words, MicroHDF can aid in the discovery of disease biomarkers.

ASD is a severe neurodevelopmental disorder whose prevalence has increased dramatically over the past several years. A recent clinical study has reported a close association between a wide range of microbes and ASD. *Prevotella* and *Firmicutes* exhibited differing abundances between the ASD and neurotypical (NT) groups, whereas *Lachnospira* shows a pattern of decreased abundance [66]. These microorganisms are ranked in the top ten list of results from our model. In addition to the microorganisms confirmed in the literature, we discover three other microorganisms, *Clostridium sensu stricto 13*, *Sulfurovum* and *Pedobacter*, which are not directly associated with ASD. There is a report that *pedobacter* and *Sulfurovum* appear to be more abundant in gastrointestinal (GI) symptoms(such as gaseousness and diarrhea) [67], which often co-occur with core ASD symptoms in children with ASD [67–69]. Their significance as microbes in ASD should be confirmed in future clinical experiments. In summary, we observe that the microbial features selected in our framework are closely associated with disease, indicating that MicroHDF can predict candidate microbes for a given disease, thus greatly assisting in the screening of candidate biomarkers.

## Discussion

Predicting host phenotypes based on microbiome data is challenging because of the substantial individual differences in the microbial community influenced by genetics, diet, lifestyle, environmental factors, and other variables. In this study, we present a novel prediction framework, MicroHDF, which is a DF-based ensemble model for inferring host phenotypes from microbial data. MicroHDF initially embed phylogenetic tree knowledge of the microbiome and the relative abundance of microbial taxa to construct a new feature matrix. Subsequently, the RF-CUS and ERTs units in the cascade layers are utilized to address class-imbalanced and high-dimensional problems, respectively. Simultaneously, the learned embeddings incorporated by the cascade layers aid in feature representation learning for the output layer. Finally, predicted labels are obtained using an ensemble strategy. Additionally, FIV is introduced to rank and quantify the contribution of microbiome features to disease prediction. A comparison with state-of-the-art methods and ablation study results confirmed that the improved deep-forest-based approach learns more discriminative features from microbes and effectively addresses metagenomic data challenges. Furthermore, the experimental results demonstrated the enhancement gained by fusing the phylogenetic tree information in our proposed approach.

MicroHDF offers the advantage of integrating multiple microbial features regardless of the sample label imbalance and feature dimension. In addition, it performs well when trained on small-to medium-scale datasets. However, our method has some limitations. First, MicroHDF exclusively utilizes microbial features and overlooks the potential of metadata (such as age, sex, BMI, and lifestyle) in host disease prediction, potentially biasing well-known microbe-disease associations. To address this issue, we can easily integrate the collected host metadata such as diet, stress, and drug frequency. These factors are generally considered confounding variables that must be addressed before model application. In metagenomic data analysis, batch effects can arise from biological factors (e.g. variations in demographics), technical factors (e.g. experiment temperatures, reagents, runs, platforms), and computational factors (e.g. pipelines, software, parameters). Biological factors can alter microbiota composition by affecting certain microorganisms, while technical factors can introduce artificial heterogeneity, and computational aspects can systematically influence all microbial variables. Therefore, to achieve more accurate predictions, it is crucial to account for and remove these confounding factors. Furthermore, the gut microbiomes associated with comorbidities may exhibit microbial patterns that are different from those linked to a single disease, potentially significantly disrupting disease prediction. Hence, we should take into account the impact of different disease correlations. Finally, the predictive model may not be generalizable to complex mental diseases, which presents an inherent challenge for disease prediction. In the future, we plan to apply more advanced deep learning models such as graph neural networks to further enhance disease prediction and explore mental disease screening.

Collectively, MicroHDF demonstrates outstanding performance and interpretability for host phenotype prediction, confirming its effectiveness through cross-validation and cross-study validation. MicroHDF can serve as a potent tool for disease prediction based on metagenomic data.

**Key Points**

- For microbiome-based supervised disease classification, classifying the host disease phenotype is still a big challenge, partly because of imbalanced classification labels and high-dimensional taxonomic abundance features in metagenomic data.
- We propose a framework with an improved DF based on the microbiome, termed MicroHDF, for host disease prediction.
- In MicroHDF, two modified forest types are embedded in the cascade layer: an RF-CUS unit dealing with data imbalance and an ERTs unit dealing with high-dimensional features.
- MicroHDF leverages the biological knowledge of microbial taxa abundance profiles through a phylogenetic tree, which improves the robustness of classification performance.

## Supplementary Material

Supplementary_revised_v4-final_bbae530

## Data Availability

The original contributions presented in this study are included in the article/supplementary material, further inquiries can be directed to the corresponding authors. The codes and datasets are available online at https://github.com/glutBiolab/MicroHDF.
