# Supplementary material for: MicroHDF: predicting host phenotypes with metagenomic data using a deep forest-based framework
Source: Brief Bioinform. 2024 Oct 24;25(6):bbae530. doi: 10.1093/bib/bbae530 (PMC11500453; doi:10.1093/bib/bbae530)
Supplement: Supplementary_revised_v4-final_bbae530 [file supplementary_revised_v4-final_bbae530.docx]

# Supplement information

## The phylogenetic tree features representation

A phylogenetic tree, also known as a phylogeny or evolutionary tree, is a graphical representation depicting the evolutionary history and relationships among a set of species or taxa over a specific period. In our work, we directly utilize PhyIoT (https://phylot.biobyte.de/) to generate phylogenetic tree based on the taxonomic annotations of microbial abundance profiles. Then, we extract the taxa phylogenetic relationship information by level traversal and postorder traversing the node on the tree. In this scenario, the postorder traversing order implies the relative temporal sequence of each taxon in tree. For level traversal, different levels on the phylogenetic tree can be treated for evolution and the fewer similarities with the ancestor. Thus, the level traversing order implies genetic information.

To incorporate the phylogenetic information more efficiently, we first use the tree as a template to construct a populated tree for each sample in the dataset. Subsequently, the value of each taxon from sample is assigned to its respective node in the tree. Finally, through level and postorder traversing the node on the tree, phylogenetically correlated taxa close to each other for each sample, forming a one-dimensional vector, which is then transformed into a matrix for the dataset.


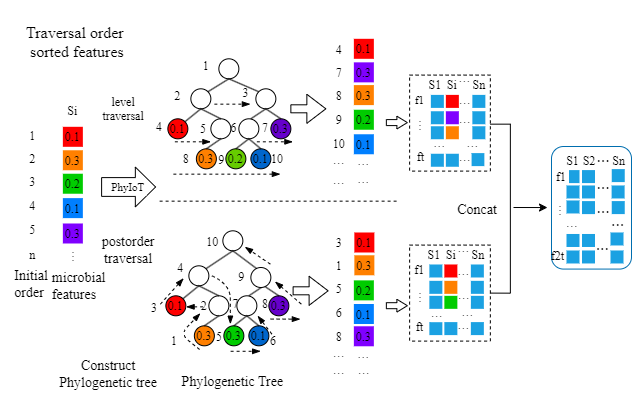


Figure S1. The flowchart for generating phylogenetic tree features

| Algorithm 1 Generating phylogenetic tree features |
| --- |
| **Input:** A set of samples where each input $M$ is a matrix representation of relative abundance  **Output:** A matrix which preserves both the abundance data and the phylogenetic informatic of the tree.  1: **For** each sample $i$to $n$ **do**  2: Generate sample $S_{i}$phylogenetic tree  3: **For** each node $k$in phylogenetic tree **do**  4: $M_{i,k}\leftarrow a_{n}$ |
| 5: Level travel phylogenetic tree with relative abundance |
| 6: $k\leftarrow k+1$ |
| 7: Post-order travel phylogenetic tree with abundance |
| 8: $k\leftarrow k+1$ |
| 9:  **End For** |
| 10: Sample $n\leftarrow$Sample$i$ |
| 11:**End For** |
| 12: Return $M^{'}$ |

$a_{n}$denote the relative abundance of the node $n$, $i,k$ denote the index of matrix $M$

## Data availability and processing

The datasets utilized in this study comprise processed microbial relative abundance data from Pasolli et al. (2016, PLoS Comput Biol) and Giliberti R (2022, PLoS Comput Biol), as well as ASD datasets processed through our analysis pipeline. The primary data processing workflow is as follows:

1. Whole-genome shotgun sequencing data analysis

For datasets such as IBD, Cirrhosis, and Obesity, Pasolli et al. [1]meticulously reprocessed the raw data following the Standard Operating Procedures (SOP) of the Human Microbiome Project. To ensure the quality of the read sequences, those shorter than 90 nucleotides were excluded. Samples not meeting these criteria were excluded from further analysis. The MetaPhlAn2 software, with default parameters, was then employed to extract species-level relative abundances from the preprocessed metagenomic samples, effectively converting the raw sequence data into a format suitable for machine learning analysis.

For NielsenHB_2014 dataset, 396 metagenomic samples were performed deep sequencing[3]. The FastX toolkit was utilized to filter 2.32 billion raw sequencing reads, resulting in the retention of 92% high-quality reads. Assembly was carried out using SOAPdenovo, generating scaftigs. Gene prediction was performed on scaftigs longer than 500 bp using MetaGeneMark. These genes were then clustered using BLAT to remove redundancy and contaminants, creating a non-redundant reference gene set. Giliberti R[2] applied MetaPhlAn3 to the processed metagenomic samples, generating species-level taxonomic profiles and extracting relative abundances at other taxonomic levels.

For IjazUz_2017 dataset, IBD metagenomic samples were performed whole-genome shotgun sequencing[4]. Trimmomatic was used to trim raw sequencing reads, removing Nextera adapters and low-quality ends. Bowtie2 was employed to align the reads to the hg18 human reference genome, discarding the matched sequences. The remaining reads were subsampled to 2 million and used to construct a gene set. Giliberti R[2] used MetaPhlAn3 for taxonomic annotation of the processed metagenomic samples, generating species-level taxonomic profiles and extracting relative abundances at other taxonomic levels.

1. 16S amplicon sequencing data analysis

The raw sequencing data of ASD (PRJNA758217, PRJNA644763, PRJNA168470, PRJNA453621) were downloaded from NCBI SRA. We used QIIME 2 version 2022.2 to deal with the raw sequences. Stitched reads were quality filtered using the quality-filter plugin and reads were denoised using DADA2 to produce amplicon sequence variants (ASVs). Taxonomic assignment of the dataset was classified against the SILVA database version 138.1, and samples with fewer than 100 reads are discarded, ASVs with fewer than 10 reads or those present in less than 1% of the samples are also removed. Species-level relative abundance results were used for subsequent analyses. To facilitate cross-validation and cross-study validation, we removed batch effects by using the R package MMUPHin.

[1] Pasolli E, Truong DT, Malik F, Waldron L, Segata N. Machine Learning Meta-analysis of Large Metagenomic Datasets: Tools and Biological Insights. PLoS Comput Biol. 2016;12(7):e1004977.

[2] Giliberti R, Cavaliere S, Mauriello IE, Ercolini D, Pasolli E. Host phenotype classification from human microbiome data is mainly driven by the presence of microbial taxa. PLoS Comput Biol. 2022;18(4):e1010066.

[3] Nielsen HB, Almeida M, Juncker AS, et al. Identification and assembly of genomes and genetic elements in complex metagenomic samples without using reference genomes. Nat Biotechnol. 2014;32(8):822-828.

[4] Ijaz UZ, Quince C, Hanske L, et al. The distinct features of microbial 'dysbiosis' of Crohn's disease do not occur to the same extent in their unaffected, genetically-linked kindred. PLoS One. 2017;12(2):e0172605.

## Parameter analysis

The number of forest units in each layer is another hyperparameter. Interestingly, similar AUC values were obtained under a single-layer structure with (RF-CUS *2 + ERTs*2) and (RF-CUS *3 + ERTs*3), whereas the single-layer configuration (RF-CUS *4 + ERTs*4) exhibited decreased performance (Table S1). This finding suggests that the overall performance of the module did not improve significantly with an increase in the number of units. We further investigated the use of BAGging (BAG) and balanced random forests (BRF) as units in the cascade. However, the model performance did not exhibit an improvement compared with that of MicroHDF. Therefore, we set the units to RF-CUS *2 and ERTs*2.

Table S1. Performance on different unit configurations of the learning module

| Datasets | Units configuration | Description | AUC(%) |
| --- | --- | --- | --- |
| Synthetic data | RF-CUS*1+ERTs*1 | Two units are placed on each cascade layer | 84.37 |
|  | RF-CUS*2+ERTs*2 | Four units are placed on each cascade layer | 89.20 |
|  | RF-CUS*3+ERTs*3 | Six units are placed on each cascade layer | 88.68 |
|  | RF-CUS*4+ERTs*4 | Eight units are placed on each cascade layer | 88.61 |
|  | RF-CUS*2+BAG*2 | Remove ERTs units replaced BAG units | 88.25 |
|  | GBC*2+ERTs*2 | Remove RF-CUS units replaced GBC units | 87.98 |
|  | RFCUS*1+ERTs*1  +BAG*1+BRF*1 | Four different units are placed on each  cascade layer | 88.62 |

## Deep forest-based unit(RF-CUS)

The deep forest-based unit(RF-CUS) is designed to deal with the imbalanced data, which leverage a class-rebalancing strategy and RF ensemble prediction. We conduct five-fold cross-validation, where in each validation fold, the training set undergoes Affinity Propagation (AP) clustering to group the majority class samples. The test set is then utilized to evaluate the model's performance. The Affinity Propagation (AP) clustering algorithm autonomously discovers cluster centers and assigns data points to clusters. The AP clustering method relies on a robust similarity matrix of the dataset, alongside a responsibility matrix R and an availability matrix A. Through iterative updates as defined in equations (1) (2) and (3), AP dynamically determines the number of clusters based on the data structure, rather than relying on a predefined number *k*.

$r_{t+1}\left( i,k \right)\leftarrow s\left( i,k \right)-{}_{k^{'}s.t.k'\neq k}^{\max}\left\{ a_{t}\left( i,k^{'} \right)+s\left( i,k^{'} \right) \right\}$ (1)

$a_{t+1}\left( i,k \right)\leftarrow\min\left\{ 0,r_{t}\left( k,k \right)+\sum_{i^{'}s.t.i^{'}\notin\left\{ i,k \right\}} \max\left\{ 0,r_{t}\left( i^{'},k \right) \right\} \right\},i\neq k.$ (2)

$a_{t+1}\left( k,k \right)=\sum_{i^{'}s.t.i^{'}\notin\left\{ i,k \right\}} \max\left\{ 0,r_{t}\left( i^{'},k \right) \right\},i=k$ (3)

In our study, we employed the Bray-Curtis dissimilarity metric for sample similarity calculations. When the initial method yields too few or too many clusters that fail to represent the underlying data structure accurately, we adjust the maximum number of iterations (max_iter) and the maximum convergence criteria (max_converce) to optimize *k*, typically resulting in values between 15 and 20 for optimal classification performance. Additionally, we implemented an AP fallback mechanism to address convergence issues. If the model fails to converge, indicated by exceeding the maximum number of iterations (commonly set to max_iter between 45 and 55) or surpassing the maximum convergence criteria (typically set to max_converce between 25 and 30), we replace the AP algorithm with K-means clustering. These measures ensure robust clustering by re-evaluating and adjusting the number of clusters based on the inherent structure of the data. Subsequently, stratified undersampling is performed within different clusters to generate subsets, with each class sampled at a specific ratio based on the size of the small sample dataset for formula (4):

$\frac{n_{cluster\_i}}{n_{majority class}}\times\left( {\lambda*n}_{minirity class} \right)，\lambda=\frac{3}{4}$ (4)

this process is repeated five times (N = 5). This results in the creation of a serial of sample sets for majority classes. Bootstrap sampling is repeatedly applied to the minority class samples to generate an equal number of sample sets. Subsequently, pairwise sampling is conducted to create class-balanced training sets from these two sequences of sample subsets. Finally, the basic RF classifier is employed on each of the balanced training sets, and the classification results are aggregated by voting to formulate the final prediction. Stratified undersampling performed through AP clustering ensures the preservation of valuable information from the majority class, while achieving a balanced merging of sampled datasets. Additionally, the RF ensemble framework effectively rebalances the training dataset to enhance the prediction accuracy.

Figure S2. The flowchart for the deep forest-based unit(RF-CUS)

Table S2 Performance comparation of K-means and AP Clustering on the Cirrhosis Dataset

| Methods | AUC(%) | AUPR(%) | Accuracy(%) | Recall(%) | F1-score(%) |
| --- | --- | --- | --- | --- | --- |
| K-means | 91.97 | 90.54 | 86.99 | 87.83 | 86.72 |
| AP clustering | 94.69 | 94.80 | 89.93 | 91.26 | 88.91 |

## Supplementary Experiments

Table S3. The result of comparison between MicroHDF and 12 baseline methods on Colorectal , C-T2D, EW-T2D and Obesity cohorts, with the top results highlighted in bold and the second ranked result underlined.

| Dataset | Methods | AUC(%) | AUPR(%) | Accuracy(%) | Recall(%) | F1-score(%) |
| --- | --- | --- | --- | --- | --- | --- |
| Colorectal  IR=1.52 | RF | 66.33 | 75.08 | 62.69 | 64.89 | 57.08 |
|  | SVM | 54.41 | 71.26 | 65.71 | 61.15 | 51.43 |
|  | LASSO | 60.86 | 75.46 | 64.28 | 61.09 | 52.48 |
|  | MetAML | 76.20 | 76.42 | 62.70 | 74.20 | 61.40 |
|  | GHMI | 69.03 | 72.56 | 64.40 | 63.14 | 59.32 |
|  | MLPNN | 57.72 | 73.77 | 56.31 | 58.13 | 52.48 |
|  | CNN1D | 56.92 | 71.27 | 58.46 | 54.17 | 58.65 |
|  | DeepMicro | 77.80 | 74.68 | **70.40** | 70.40 | 65.39 |
|  | Deep Forest | 70.43 | 72.33 | 65.33 | 65.42 | 67.31 |
|  | PopPhy-CNN | 62.83 | 65.23 | 60.13 | 61.76 | 62.26 |
|  | GNPI | 63.47 | 66.73 | 66.67 | 66.67 | 66.84 |
|  | GDmicro | **77.84** | **79.11** | 69.02 | **77.02** | 69.77 |
|  | MicroHDF | 76.64 | 77.90 | 67.77 | 76.44 | **69.81** |
| C-T2D  IR=1.02 | RF | 74.80 | 72.35 | 67.18 | 66.70 | 67.20 |
|  | SVM | 61.18 | 57.82 | 59.74 | 58.49 | 58.19 |
|  | LASSO | 70.35 | 68.52 | 65.77 | 64.29 | 63.91 |
|  | MetAML | 74.40 | 72.00 | 61.20 | 60.80 | 60.70 |
|  | GHMI | 71.56 | 72.91 | 64.81 | 62.45 | 65.95 |
|  | MLPNN | 72.75 | 70.61 | 62.56 | 66.01 | 65.23 |
|  | CNN1D | 65.01 | 63.66 | 59.04 | 57.61 | 66.89 |
|  | DeepMicro | 74.04 | 71.89 | 65.22 | 64.11 | 64.61 |
|  | Deep Forest | 71.91 | 72.55 | 62.78 | 60.00 | 63.37 |
|  | PopPhy-CNN | 72.06 | 69.20 | 61.20 | 63.64 | 62.90 |
|  | GNPI | 68.06 | 71.39 | 64.77 | 61.70 | 64.74 |
|  | GDmicro | **79.39** | 74.12 | **73.28** | **69.31** | 66.77 |
|  | MicroHDF | 78.96 | **75.92** | 68.86 | 67.89 | **67.31** |
| EW-T2D  IR=1.23 | RF | 65.12 | 64.25 | 72.77 | 67.77 | 67.28 |
|  | SVM | 61.03 | 60.67 | 57.22 | 62.77 | 59.30 |
|  | LASSO | 53.30 | 58.14 | 56.11 | 53.33 | 56.12 |
|  | MetAML | 65.10 | 69.80 | 59.80 | 59.50 | 58.10 |
|  | GHMI | 64.49 | 66.83 | 57.26 | 64.14 | 61.30 |
|  | MLPNN | 55.18 | 64.88 | 56.22 | 63.55 | 61.12 |
|  | CNN1D | 62.74 | 67.25 | 55.00 | 59.11 | 57.46 |
|  | DeepMicro | 72.76 | 72.00 | 68.00 | 64.58 | 70.30 |
|  | Deep Forest | 67.24 | 69.32 | 60.13 | 61.76 | 64.42 |
|  | PopPhy-CNN | 57.58 | 72.02 | 61.00 | 64.77 | 63.96 |
|  | GNPI | 69.30 | 74.17 | 61.70 | 61.70 | 61.64 |
|  | GDmicro | 72.22 | 73.38 | **74.55** | **69.99** | 70.52 |
|  | MicroHDF | **73.18** | **74.83** | 73.89 | 69.72 | **70.57** |
| Obesity  IR=1.84 | RF | 64.98 | 59.03 | 64.00 | 64.41 | 64.95 |
|  | SVM | 62.50 | 58.25 | 62.09 | 62.06 | 61.71 |
|  | LASSO | 57.24 | 58.79 | 64.41 | 65.23 | 63.43 |
|  | MetAML | 65.49 | 62.79 | 61.60 | 61.20 | 60.20 |
|  | GHMI | 62.36 | 64.41 | 62.50 | 65.41 | 63.60 |
|  | MLPNN | 56.57 | 58.46 | 62.00 | 59.45 | 58.02 |
|  | CNN1D | 61.98 | 59.47 | 62.08 | 62.14 | 62.33 |
|  | DeepMicro | 66.50 | 67.99 | 64.71 | 63.94 | 74.50 |
|  | Deep Forest | 62.96 | 62.65 | 65.47 | 66.33 | 65.35 |
|  | PopPhy-CNN | 60.10 | 61.80 | 65.10 | 66.85 | 69.08 |
|  | GNPI | 64.01 | 65.96 | 62.51 | 62.51 | 69.83 |
|  | GDmicro | 69.28 | 74.09 | **70.16** | **76.13** | 77.17 |
|  | MicroHDF | **69.70** | **75.18** | 65.23 | 68.52 | **77.22** |

Table S4. The comparison results of the Gutbalance method on two disease cohorts

|  | Datasets | AUC | AUPR |
| --- | --- | --- | --- |
| Inflammatory Bowel disease | IBD | 0.6299 | 0.4585 |
|  | NielsenHB_2014 | 0.5504 | 0.6713 |
|  | ICDF | 0.2042 | 0.3754 |
|  | IjazUz_2017 | 0.1821 | 0.5026 |
| Autism spectrum disorder | Li_ASD | 0.5341 | 0.9660 |
|  | Chen_ASD | 0.5462 | 0.6545 |
|  | Arizo_ASD | 0.5217 | 0.5053 |
|  | Dan_ASD | 0.5356 | 0.5299 |

Table S5. Friedman test comparison of all methods based on AUC.

|  | Cirrhosis | IBD | Colorectal | C-T2D | EW-T2D | Obesity | Average  rank |
| --- | --- | --- | --- | --- | --- | --- | --- |
| RF | 93.35_（3）_ | 87.36_（5）_ | 66.33_（7）_ | 74.8_（3）_ | 65.12_（6）_ | 64.98_（5）_ | 4.83 |
| SVM | 93.2_（4）_ | 75.97_（13）_ | 54.41_（13）_ | 61.18_（13）_ | 61.03_（10）_ | 62.5_（8）_ | 10.16 |
| LASSO | 88.95_（12）_ | 76.52_（12）_ | 60.86_（10）_ | 70.35_（10）_ | 53.3_（13）_ | 57.24_（12）_ | 11.50 |
| MetAML | 92.19_（6）_ | 88.2_（3）_ | 76.2_（4）_ | 74.4_（4）_ | 65.1_（7）_ | 65.49_（4）_ | 4.67 |
| GHMI | 90.51_（11）_ | 85.74_（6）_ | 69.03_（6）_ | 71.56_（9）_ | 64.49_（8）_ | 62.36_（9）_ | 8.17 |
| MLPNN | 92.14_（7）_ | 77.83_（11）_ | 57.72_（11）_ | 72.75_（6）_ | 55.18_（12）_ | 56.57_（13）_ | 10.00 |
| CNN1D | 90.57_（9）_ | 84.64_（8）_ | 56.92_（12）_ | 65.01_（12）_ | 62.74_（9）_ | 61.98_（10）_ | 10.00 |
| DeepMicro | 88.5_（13）_ | 85_（7）_ | 77.8_（2）_ | 74.04_（5）_ | 72.76_（2）_ | 66.5_（3）_ | 5.30 |
| Deep Forest | 92.03_（8）_ | 84.3_（9）_ | 70.43_（5）_ | 71.91_（8）_ | 67.24_（5）_ | 62.96_（7）_ | 7.00 |
| PopPhy-CNN | 90.53_（10）_ | 83.5_（10）_ | 62.83_（9）_ | 72.06_（7）_ | 57.58_（11）_ | 60.1_（11）_ | 9.67 |
| GNPI | 92.23_（5）_ | 87.53_（4）_ | 63.47_（8）_ | 68.06_（11）_ | 69.3_（4）_ | 64.01_（6）_ | 6.33 |
| GDmicro | 94.63_（2）_ | 88.58_（2）_ | 77.84_（1）_ | 79.39_（1）_ | 72.22_（3）_ | 69.28_（2）_ | 1.83 |
| MicroHDF | 94.69_（1）_ | 91.82_（1）_ | 76.64_（3）_ | 78.96_（2）_ | 73.18_（1）_ | 69.7_（1）_ | 1.50 |

Table S6. Friedman test comparison of all methods based on AUPR.

|  | Cirrhosis | IBD | Colorectal | C-T2D | EW-T2D | Obesity | Average  rank |
| --- | --- | --- | --- | --- | --- | --- | --- |
| RF | 93.28_（5）_ | 75.21_（5）_ | 75.08_（5）_ | 72.35_（5）_ | 64.25_（11）_ | 59.03_（10）_ | 6.83 |
| SVM | 92.28_（7）_ | 61.66_（12）_ | 71.26_（11）_ | 57.82_（13）_ | 60.67_（12）_ | 58.25_（13）_ | 11.33 |
| LASSO | 88.8_（13）_ | 58.96_（13）_ | 75.46_（4）_ | 68.52_（11）_ | 58.14_（13）_ | 58.79_（11）_ | 10.83 |
| MetAML | 93.55_（4）_ | 71.55_（9）_ | 76.42_（3）_ | 72_（6）_ | 69.8_（6）_ | 62.79_（6）_ | 5.67 |
| GHMI | 89.01_（12）_ | 76.28_（4）_ | 72.56_（8）_ | 72.91_（3）_ | 66.83_（9）_ | 64.41_（5）_ | 6.83 |
| MLPNN | 92.13_（8）_ | 72.4_（8）_ | 73.77_（7）_ | 70.61_（9）_ | 64.88_（10）_ | 58.46_（12）_ | 9.00 |
| CNN1D | 90.48_（11）_ | 66.76_（11）_ | 71.27_（10）_ | 63.66_（12）_ | 67.25_（8）_ | 59.47_（9）_ | 10.17 |
| DeepMicro | 93.2_（6）_ | 74.2_（6）_ | 74.68_（6）_ | 71.89_（7）_ | 72_（5）_ | 67.99_（3）_ | 5.50 |
| Deep Forest | 91.13_（10）_ | 76.35_（3）_ | 72.33_（9）_ | 72.55_（4）_ | 69.32_（7）_ | 62.65_（7）_ | 6.67 |
| PopPhy-CNN | 91.4_（9）_ | 70_（10）_ | 65.23_（13）_ | 69.2_（10）_ | 72.02_（4）_ | 61.8_（8）_ | 9.00 |
| GNPI | 93.87_（2）_ | 73.12_（7）_ | 66.73_（12）_ | 71.39_（8）_ | 74.17_（2）_ | 65.96_（4）_ | 5.83 |
| GDmicro | 93.82_（3）_ | 78.89_（2）_ | 79.11_（1）_ | 74.12_（2）_ | 73.38_（3）_ | 74.09_（2）_ | 2.17 |
| MicroHDF | 94.8_（1）_ | 79.62_（1）_ | 77.9_（2）_ | 75.92_（1）_ | 74.83_（1）_ | 75.18_（1）_ | 1.17 |

Table S7. Comparison results of the influence of phylogenetic tree information. Raw feature (O) notes that the model used raw data, raw feature (L), raw feature (P), and raw feature (L+P) present that the model generated the new taxa representations by traversing the phylogenetic tree with level traversal, postorder traversal, and level traversal combined with depth traversal.

| Dataset | feature | AUC(%) | AUPR(%) | Accuracy(%) | Recall(%) | F1-score(%) |
| --- | --- | --- | --- | --- | --- | --- |
| IBD | Raw feature(O) | 89.88 | 79.48 | 83.62 | 86.47 | 88.11 |
|  | Raw feature(O+L) | 91.29 | 78.70 | 85.45 | 84.42 | 88.93 |
|  | Raw feature(O+P) | 91.29 | 74.41 | 85.45 | 84.42 | 88.96 |
|  | Raw feature(O+L+P) | **91.82** | **79.62** | **86.35** | **86.49** | **89.59** |
| Obesity | Raw feature(O) | 68.09 | 73.21 | 62.23 | 64.25 | 75.62 |
|  | Raw feature(O+L) | 68.93 | 74.52 | 64.62 | 66.24 | 76.73 |
|  | Raw feature(O+P) | 69.06 | 74.74 | 64.20 | 67.08 | 76.76 |
|  | Raw feature(O+L+P) | **69.70** | **75.18** | **65.23** | **68.52** | **77.22** |
| Colorectal | Raw feature(O) | 73.90 | 75.82 | 63.66 | 69.56 | 67.05 |
|  | Raw feature(O+L) | 74.62 | 77.12 | 65.27 | 74.22 | 68.75 |
|  | Raw feature(O+P) | 75.01 | **77.98** | 67.77 | 76.44 | 68.80 |
|  | Raw feature(O+L+P) | **76.64** | 77.90 | **67.77** | **76.44** | **69.81** |
| EW-T2D | Raw feature(O) | 68.09 | 72.26 | 71.89 | 67.33 | 68.68 |
|  | Raw feature(O+L) | 72.12 | 73.59 | 73.79 | 69.50 | 69.97 |
|  | Raw feature(O+P) | 72.88 | 73.75 | 73.79 | 69.50 | 69.84 |
|  | Raw feature(O+L+P) | **73.18** | **74.83** | **73.89** | **69.72** | **70.57** |
| Cirrhosis | Raw feature(O) | 94.68 | 93.93 | 89.93 | 89.45 | 84.88 |
|  | Raw feature(O+L) | 94.69 | 93.93 | 88.93 | 93.00 | 87.35 |
|  | Raw feature(O+P) | **95.52** | 93.94 | 89.80 | 91.23 | 87.94 |
|  | Raw feature(O+L+P) | 94.96 | **94.80** | **89.93** | **91.26** | **88.91** |
| C-T2D | Raw feature(O) | 74.86 | 73.92 | 65.86 | 64.89 | 65.31 |
|  | Raw feature(O+L) | 76.96 | 74.18 | 67.19 | 67.07 | 66.00 |
|  | Raw feature(O+P) | 76.42 | 75.34 | 67.19 | 67.10 | 66.53 |
|  | Raw feature(O+L+P) | **78.96** | **75.92** | **68.86** | **67.89** | **67.31** |
| Li_ASD | Raw feature(O) | 76.48 | 74.33 | 85.53 | 76.58 | 68.98 |
|  | Raw feature(O+L) | 78.48 | 75.58 | 86.33 | 77.12 | 71.73 |
|  | Raw feature(O+P) | 78.96 | 76.66 | 86.35 | 77.89 | 71.22 |
|  | Raw feature(O+L+P) | **80.66** | **79.37** | **86.35** | **78.00** | **71.90** |

Table S8. Performance comparison of different models using a composite matrix concatenating abundance data across all hierarchical levels

| Dataset | Methods | AUC(%) | AUPR(%) | Accuracy(%) | Recall(%) | F1-score(%) |
| --- | --- | --- | --- | --- | --- | --- |
| Cirrhosis  IR=1.03 | RF | 94.19 | 93.42 | 88.50 | 90.13 | 87.77 |
|  | SVM | 93.66 | 93.32 | 84.08 | 87.82 | 87.80 |
|  | LASSO | 89.37 | 89.86 | 88.15 | 82.71 | 79.74 |
|  | MetAML | 93.81 | 94.30 | 88.35 | 85.84 | 87.82 |
|  | GHMI | 92.51 | 91.74 | 83.17 | 87.80 | 83.61 |
|  | MLPNN | 92.48 | 92.77 | 82.06 | 89.93 | 87.96 |
|  | CNN1D | 88.33 | 89.04 | 84.92 | 84.67 | 85.06 |
|  | DeepMicro | 89.40 | 93.62 | 83.72 | 76.83 | 82.27 |
|  | Deep Forest | 93.56 | 93.59 | 87.52 | 87.52 | 87.48 |
|  | PopPhy-CNN | ---- | ---- | ---- | ---- | ---- |
|  | GNPI | ---- | ---- | ---- | ---- | ---- |
|  | GDmicro | 94.34 | 92.00 | **91.39** | **94.77** | **89.55** |
|  | MicroHDF- S | 94.41 | 93.85 | 87.93 | 90.32 | 88.04 |
|  | MicroHDF- T | **94.69** | **94.80** | 89.93 | 91.26 | 88.91 |
| IBD  IR=3.4 | RF | 87.65 | 74.41 | 85.00 | 82.82 | 76.44 |
|  | SVM | 76.76 | 66.52 | 82.27 | 80.00 | 73.18 |
|  | LASSO | 78.66 | 61.93 | 79.88 | 78.71 | 70.77 |
|  | MetAML | 90.48 | 78.07 | 82.25 | 76.96 | 79.54 |
|  | GHMI | 87.25 | 79.48 | 84.55 | 87.35 | 89.41 |
|  | MLPNN | 84.59 | 77.39 | 82.73 | 84.71 | 83.51 |
|  | CNN1D | 87.17 | 70.13 | 84.91 | 79.42 | 73.56 |
|  | DeepMicro | 84.82 | 72.24 | 83.56 | 78.36 | 81.52 |
|  | Deep Forest | 87.90 | 79.53 | 80.00 | 80.00 | 74.72 |
|  | PopPhy-CNN | ---- | ---- | ---- | ---- | ---- |
|  | GNPI | ---- | ---- | ---- | ---- | ---- |
|  | GDmicro | 87.29 | **87.49** | **87.73** | **90.59** | 88.67 |
|  | MicroHDF- S | 89.00 | 76.00 | 84.73 | 84.47 | 88.59 |
|  | MicroHDF- T | **91.82** | 79.62 | 86.35 | 86.49 | **89.59** |
| Colorectal  IR=1.52 | RF | 65.98 | 74.78 | 64.60 | 65.56 | 60.17 |
|  | SVM | 56.26 | 73.51 | 62.20 | 64.00 | 55.30 |
|  | LASSO | 67.01 | 71.50 | 62.73 | 65.50 | 62.24 |
|  | MetAML | 76.44 | 76.59 | 64.60 | 76.09 | 67.51 |
|  | GHMI | 73.48 | 75.70 | 69.33 | 65.01 | 63.38 |
|  | MLPNN | 59.60 | 74.24 | 61.80 | 62.24 | 64.03 |
|  | CNN1D | 58.48 | 73.99 | 60.27 | 58.05 | 62.07 |
|  | DeepMicro | 77.33 | 75.11 | 71.02 | 71.33 | 66.75 |
|  | Deep Forest | 72.75 | 76.05 | 66.13 | 69.62 | 65.69 |
|  | PopPhy-CNN | ---- | ---- | ---- | ---- | ---- |
|  | GNPI | ---- | ---- | ---- | ---- | ---- |
|  | GDmicro | **82.19** | **79.29** | **74.33** | 68.86 | **75.41** |
|  | MicroHDF- S | 74.16 | 77.19 | 64.57 | 75.61 | 67.72 |
|  | MicroHDF- T | 76.64 | 77.90 | 67.77 | **76.44** | 69.81 |
| C-T2D  IR=1.02 | RF | 75.26 | 72.69 | 68.00 | 67.35 | 67.07 |
|  | SVM | 63.31 | 61.72 | 61.60 | 60.81 | 61.04 |
|  | LASSO | 74.99 | 72.52 | 67.45 | 65.51 | 65.74 |
|  | MetAML | 75.31 | **75.98** | 68.31 | 66.47 | 67.49 |
|  | GHMI | 74.84 | 72.78 | 67.15 | 65.45 | 66.43 |
|  | MLPNN | 72.63 | 74.05 | 67.44 | 68.95 | 67.40 |
|  | CNN1D | 70.46 | 70.60 | 64.53 | 62.02 | **68.00** |
|  | DeepMicro | 78.32 | 73.29 | 68.53 | 66.43 | 66.21 |
|  | Deep Forest | 76.48 | 75.10 | 68.33 | 62.35 | 65.58 |
|  | PopPhy-CNN | ---- | ---- | ---- | ---- | ---- |
|  | GNPI | ---- | ---- | ---- | ---- | ---- |
|  | GDmicro | 73.22 | 75.93 | **71.52** | **74.28** | 67.82 |
|  | MicroHDF- S | 78.33 | 74.85 | 67.45 | 66.08 | 65.55 |
|  | MicroHDF-T | **78.96** | 75.92 | 68.86 | 67.89 | 67.31 |
| EW-T2D  IR=1.23 | RF | 71.95 | 72.54 | 74.74 | 69.17 | 70.57 |
|  | SVM | 62.82 | 61.51 | 60.47 | 65.83 | 61.04 |
|  | LASSO | 58.93 | 62.84 | 62.93 | 68.71 | 63.64 |
|  | MetAML | 71.40 | 73.93 | 67.79 | 66.98 | 66.00 |
|  | GHMI | 72.67 | 72.79 | 63.47 | 69.87 | 66.25 |
|  | MLPNN | 65.47 | 68.05 | 65.47 | 72.00 | 68.99 |
|  | CNN1D | 67.60 | 70.87 | 60.68 | 66.78 | 60.33 |
|  | DeepMicro | 71.31 | 73.54 | 70.00 | 68.82 | 69.89 |
|  | Deep Forest | 72.28 | 74.13 | 69.93 | 70.32 | 69.97 |
|  | PopPhy-CNN | ---- | ---- | ---- | ---- | ---- |
|  | GNPI | ---- | ---- | ---- | ---- | ---- |
|  | GDmicro | **79.30** | 65.57 | **73.95** | **78.06** | **74.57** |
|  | MicroHDF- S | 71.20 | 72.14 | 72.95 | 68.06 | 68.12 |
|  | MicroHDF- T | 73.18 | **74.83** | 73.89 | 69.72 | 70.57 |
| Obesity  IR=1.84 | RF | 65.33 | 62.60 | 66.82 | 68.04 | 66.56 |
|  | SVM | 64.92 | 62.31 | 64.41 | 64.07 | 62.51 |
|  | LASSO | 59.70 | 60.87 | 65.31 | 66.00 | 67.00 |
|  | MetAML | 64.62 | 74.51 | 64.43 | 65.82 | 76.93 |
|  | GHMI | 62.70 | 68.94 | 64.92 | 66.39 | 65.29 |
|  | MLPNN | 56.89 | 70.41 | 65.62 | 70.42 | 76.04 |
|  | CNN1D | 62.42 | **75.38** | 63.26 | 72.33 | 72.95 |
|  | DeepMicro | 66.33 | 73.97 | 68.63 | 72.84 | 78.49 |
|  | Deep Forest | 61.97 | 72.96 | 64.02 | **73.79** | 62.61 |
|  | PopPhy-CNN | ---- | ---- | ---- | ---- | ---- |
|  | GNPI | ---- | ---- | ---- | ---- | ---- |
|  | GDmicro | **70.44** | 72.02 | **70.39** | 68.63 | 69.99 |
|  | MicroHDF- S | 65.35 | 73.78 | 64.05 | 66.73 | 73.43 |
|  | MicroHDF- T | 69.70 | 75.18 | 65.23 | 68.52 | **77.22** |

Note: The methods PopPhy-CNN and GNPI, which specifically depend on phylogenetic tree information, are not applicable to data aggregated across all hierarchical levels. MicroHDF-S refers to our model running on aggregated data matrices without incorporating phylogenetic tree information, while MicroHDF-T indicates our model operating on species-level abundance data integrated with phylogenetic tree information.

Table S9. Performance comparison of two model architectures, the single channel, which denotes microbial taxa features, and phylogenetic tree knowledge are combined as the input of the model. Two channels denote that microbial taxonomic features and phylogenetic tree knowledge are fed into the model.

| Dataset | Architectures | AUC (%) | AUPR (%) | Accuracy (%) | Recall (%) | F1-score (%) |
| --- | --- | --- | --- | --- | --- | --- |
| IBD | Single channel | 90.82 | 79.48 | 84.52 | **86.62** | 87.99 |
|  | Two channels | **91.82** | **79.62** | **86.35** | 86.49 | **89.59** |
| Obesity | Single channel | 69.22 | 73.74 | 63.22 | 67.55 | 71.80 |
|  | Two channels | **69.70** | **75.18** | **65.23** | **68.52** | **77.22** |
| Colorectal | Single channel | 71.64 | 76.98 | 65.56 | **76.44** | 68.95 |
|  | Two channels | **76.64** | **77.90** | **67.77** | **76.44** | **69.81** |
| EW-T2D | Single channel | 72.01 | 72.76 | 71.89 | 68.83 | 68.96 |
|  | Two channels | **73.18** | **74.83** | **73.89** | **69.72** | **70.57** |
| Cirrhosis | Single channel | 94.68 | 94.19 | 87.95 | 90.49 | 88.67 |
|  | Two channels | **94.69** | **94.80** | **89.93** | **91.26** | **88.91** |
| C-T2D | Single channel | 78.76 | 75.50 | 67.41 | 66.79 | **67.62** |
|  | Two channels | **78.96** | **75.92** | **68.86** | **67.89** | 67.31 |
| Li_ASD | Single channel | 78.87 | 76.50 | 83.37 | 77.69 | 70.00 |
|  | Two channels | **80.66** | **79.37** | **86.35** | **78.00** | **71.90** |

Table S10. Prediction results of the top 50 IBD-associated microbes.

| Disease | Rank | FIV | LDA score | Microbiota |
| --- | --- | --- | --- | --- |
| IBD | 1 | 0.2818 | 3.252 | Unclassified Oscillibacter |
|  | 2 | 0.2364 | 3.427 | Bacteroides intestinalis |
|  | 3 | 0.1745 | 3.165 | Odoribacter splanchnicus |
|  | 4 | 0.1545 | 3.244 | Ruminococcus lactaris |
|  | 5 | 0.1364 | 2.990 | Roseburia hominis |
|  | 6 | 0.1091 | 3.210 | Bifidobacterium bifidum |
|  | 7 | 0.1091 | 3.201 | Alistipes finegoldii |
|  | 8 | 0.1090 | 3.171 | Ruminococcus bromii |
|  | 9 | 0.1000 | 2.777 | Lachnospiracea bacterium 8_1_57FAA |
|  | 10 | 0.0818 | 2.689 | Bacteroides vulgatus |
|  | 11 | 0.0808 | 2.620 | Lachnospiracea bacterium 8_1_58FAA |
|  | 12 | 0.0799 | 2.611 | Akkermansia muciniphila |
|  | 13 | 0.0784 | 2.602 | Faecalibacterium prausnitzii |
|  | 14 | 0.0784 | 2.597 | Alistipes shahii |
|  | 15 | 0.0771 | 2.597 | Bacteroides cellulosilyticus |
|  | 16 | 0.0769 | 2.577 | Coprococcus comes |
|  | 17 | 0.0768 | 2.564 | Odoribacter_unclassified |
|  | 18 | 0.0767 | 2.537 | Butyrivibrio_crossotus |
|  | 19 | 0.0757 | 2.476 | Coprococcus_sp_ART55_1 |
|  | 20 | 0.0756 | 2.426 | Barnesiella_intestinihominis |
|  | 21 | 0.0752 | 2.348 | Ruminococcus_lactaris |
|  | 22 | 0.0751 | 2.341 | Eubacterium_ventriosum |
|  | 23 | 0.0751 | 2.332 | Bifidobacterium_animalis |
|  | 24 | 0.0748 | 2.241 | Erysipelotrichaceae_bacterium_3_1_53 |
|  | 25 | 0.0746 | 2.218 | Proteus_mirabilis |
|  | 26 | 0.0744 | 2.207 | unclassified_Rothia |
|  | 27 | 0.0742 | 2.205 | Bacteroides_barnesiae |
|  | 28 | 0.0739 | 2.203 | Akkermansia_muciniphila |
|  | 29 | 0.0733 | 1.867 | Gardnerella_vaginalis |
|  | 30 | 0.0730 | 1.828 | Bacteroides_caccae |
|  | 31 | 0.0729 | 1.828 | Bacteroides_coprophilus |
|  | 32 | 0.0727 | 1.792 | Citrobacter_freundii |
|  | 33 | 0.0725 | 1.775 | Adlercreutzia_equolifaciens |
|  | 34 | 0.0721 | 1.576 | Collinsella_unclassified |
|  | 35 | 0.0720 | 1.736 | Enterobacteriaceae_bacterium_9_2_54FAA |
|  | 36 | 0.0693 | 1.713 | Bacteroides_cellulosilyticus |
|  | 37 | 0.0690 | 1.703 | unclassified_Odoribacter |
|  | 38 | 0.0685 | 1.621 | Odoribacter_linens |
|  | 39 | 0.0665 | 1.611 | Enterobacter_aerogenes |
|  | 40 | 0.0658 | 1.515 | Bacteroides_clarus |
|  | 41 | 0.0642 | 1.515 | Citrobacter_unclassified |
|  | 42 | 0.0600 | 1.467 | Butyrivibrio_unclassified |
|  | 43 | 0.0596 | 1.466 | Parabacteroides_distasonis |
|  | 44 | 0.0587 | 1.455 | Brevibacterium_linens |
|  | 45 | 0.0573 | 1.387 | Bacteroides_sp_1_1_14 |
|  | 46 | 0.0560 | 1.337 | Bacteroides_sp_1_1_30 |
|  | 47 | 0.0541 | 1.336 | Porphyromonas_asaccharolytica |
|  | 48 | 0.0512 | 1.315 | Parabacteroides_unclassified |
|  | 49 | 0.0506 | 1.241 | Parabacteroides_goldsteinii |
|  | 50 | 0.0495 | 1.236 | Odoribacter_laneus |

Table S11. Prediction results of the top 50 ASD-associated microbes.

| Disease | Rank | FIV | LDA score | Microbiota |
| --- | --- | --- | --- | --- |
| ASD | 1 | 0.0576 | 4.524 | Eubacterium limosum |
|  | 2 | 0.0575 | 4.503 | Ruminococcaceae UCG-003 |
|  | 3 | 0.0563 | 4.498 | Clostridium sensu stricto 13 |
|  | 4 | 0.0533 | 4.441 | Prevotella |
|  | 5 | 0.0533 | 4.441 | Lachnospiraceae_NK4A136 |
|  | 6 | 0.0532 | 4.389 | Lachnospira |
|  | 7 | 0.0531 | 4.328 | Clostridium perfringens |
|  | 8 | 0.0530 | 4.328 | Bifdobacterium |
|  | 9 | 0.0512 | 4.220 | Sulfurovum |
|  | 10 | 0.0503 | 4.216 | Pedobacter |
|  | 11 | 0.0500 | 4.206 | Bacteroides_uniformis |
|  | 12 | 0.0497 | 4.191 | Ruminococcus_bicirculans.1 |
|  | 13 | 0.0496 | 4.179 | Blautia glucerasea |
|  | 14 | 0.0496 | 4.143 | Alistipes_putredinis.1 |
|  | 15 | 0.0496 | 4.124 | Bacteroides_stercoris |
|  | 16 | 0.0486 | 4.024 | Bacteroides_uniformis.2 |
|  | 17 | 0.0485 | 4.003 | Bacteroides_vulgatus8 |
|  | 18 | 0.0485 | 4.002 | Alistipes_putredinis.2 |
|  | 19 | 0.0482 | 4.010 | Bacteroides_vulgatus11 |
|  | 20 | 0.0479 | 4.122 | Akkermansia muciniphila |
|  | 21 | 0.0479 | 4.000128 | Megamonas_funiformis2 |
|  | 22 | 0.0473 | 3.995545 | uncultured_Faecalibacterium.2 |
|  | 23 | 0.0473 | 3.980685 | Alistipes timonensis |
|  | 24 | 0.0473 | 3.970685 | Bacteroides ovatus |
|  | 25 | 0.0473 | 3.958011 | Coprococcus comes |
|  | 26 | 0.0472 | 3.936443 | Clostridium ventriculi |
|  | 27 | 0.0471 | 3.89216 | Ruminococcus bromii |
|  | 28 | 0.0470 | 3.870806 | [Eubacterium] rectale |
|  | 29 | 0.0464 | 3.847019 | Prevotella oryzae |
|  | 30 | 0.0461 | 3.842325 | Dorea longicatena |
|  | 31 | 0.0454 | 3.802325 | Odoribacter splanchnicus |
|  | 32 | 0.0453 | 3.782325 | [Eubacterium] sulci |
|  | 33 | 0.0450 | 3.753666 | Bacteroides_ovatus.3 |
|  | 34 | 0.0447 | 3.743353 | [Clostridium] spiroforme |
|  | 35 | 0.0447 | 3.730203 | Sporobacter termitidis |
|  | 36 | 0.0446 | 3.728988 | Alistipes onderdonkii |
|  | 37 | 0.0446 | 3.628988 | Bifidobacterium pseudocatenulatum |
|  | 38 | 0.0445 | 3.628988 | Ruminococcus champanellensis |
|  | 39 | 0.0444 | 3.603037 | Bacteroides coprocola |
|  | 40 | 0.0438 | 3.675184 | Clostridium tertium |
|  | 41 | 0.0437 | 3.629517 | Bacteroides massiliensis |
|  | 42 | 0.0435 | 3.607904 | Christensenella minuta |
|  | 43 | 0.0435 | 3.564904 | Streptococcus salivarius |
|  | 44 | 0.0430 | 3.479694 | Desulfovibrio fairfieldensis |
|  | 45 | 0.0428 | 3.428904 | Gordonibacter pamelaeae |
|  | 46 | 0.0426 | 3.422904 | [Eubacterium] siraeum |
|  | 47 | 0.0418 | 3.419904 | Dialister_invisus |
|  | 48 | 0.0404 | 3.413299 | Bacteroides_galacturonicus.4 |
|  | 49 | 0.0400 | 3.403299 | Gemmiger_formicilis.2 |
|  | 50 | 0.0400 | 3.383299 | uncultured_Faecalibacterium1 |

Table S12. Performance comparison of our method at different taxonomic ranks.

| Dataset | Phylum  AUC | Class  AUC | Order  AUC | Family  AUC | Genus  AUC | Species  AUC |
| --- | --- | --- | --- | --- | --- | --- |
| IBD | 0.8267 | 0.8362 | 0.8556 | 0.8752 | 0.8829 | **0.9133** |
| Obesity | 0.6500 | 0.6562 | 0.6681 | 0.6742 | 0.6866 | **0.6970** |
| Colorectal | 0.6449 | 0.6585 | 0.6732 | 0.7083 | 0.7346 | **0.7579** |
| EW-T2D | 0.6520 | 0.6614 | 0.6891 | 0.6960 | 0.7042 | **0.7224** |
| Cirrhosis | 0.8464 | 0.8673 | 0.9076 | 0.9216 | 0.9361 | **0.9469** |
| C-T2D | 0.7337 | 0.7433 | 0.7680 | 0.7694 | 0.7746 | **0.7882** |
| Li_ASD | 0.7042 | 0.7298 | 0.7639 | 0.7727 | 0.7926 | **0.8056** |


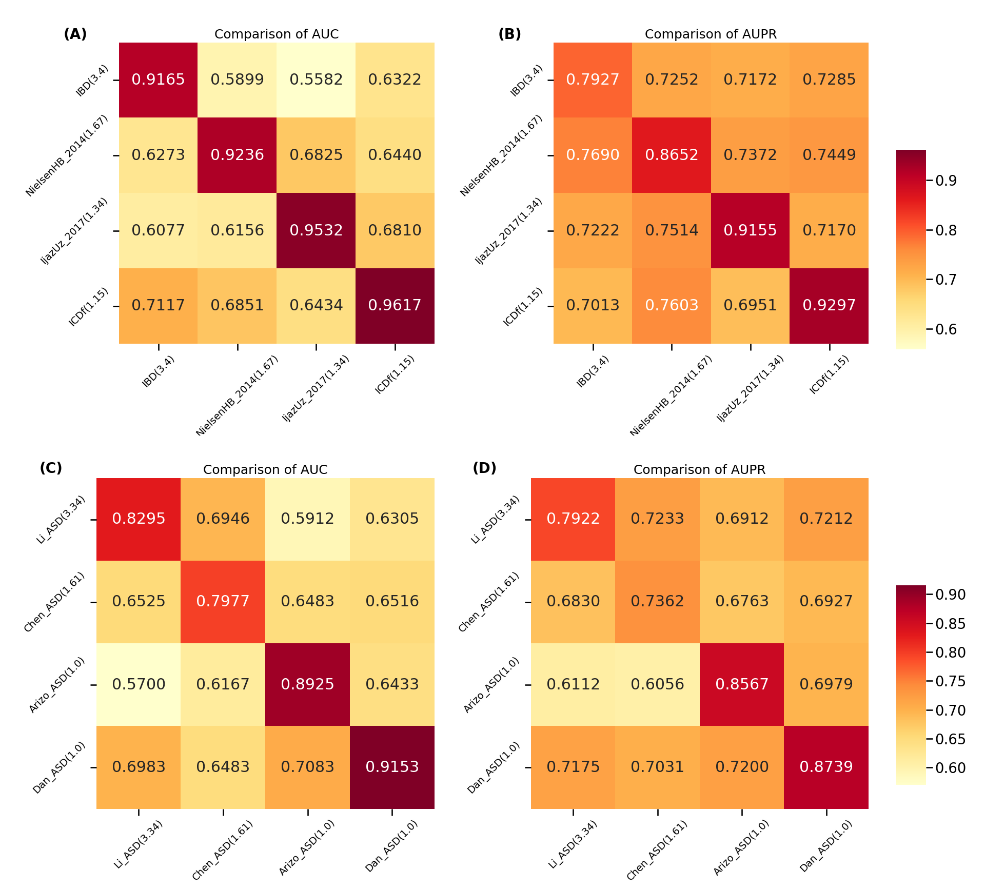


Figure S3 The performance comparison of MicroHDF across multiple cohorts for the same disease(A-B) inflammatory bowel disease (C-D) autism spectrum disorders


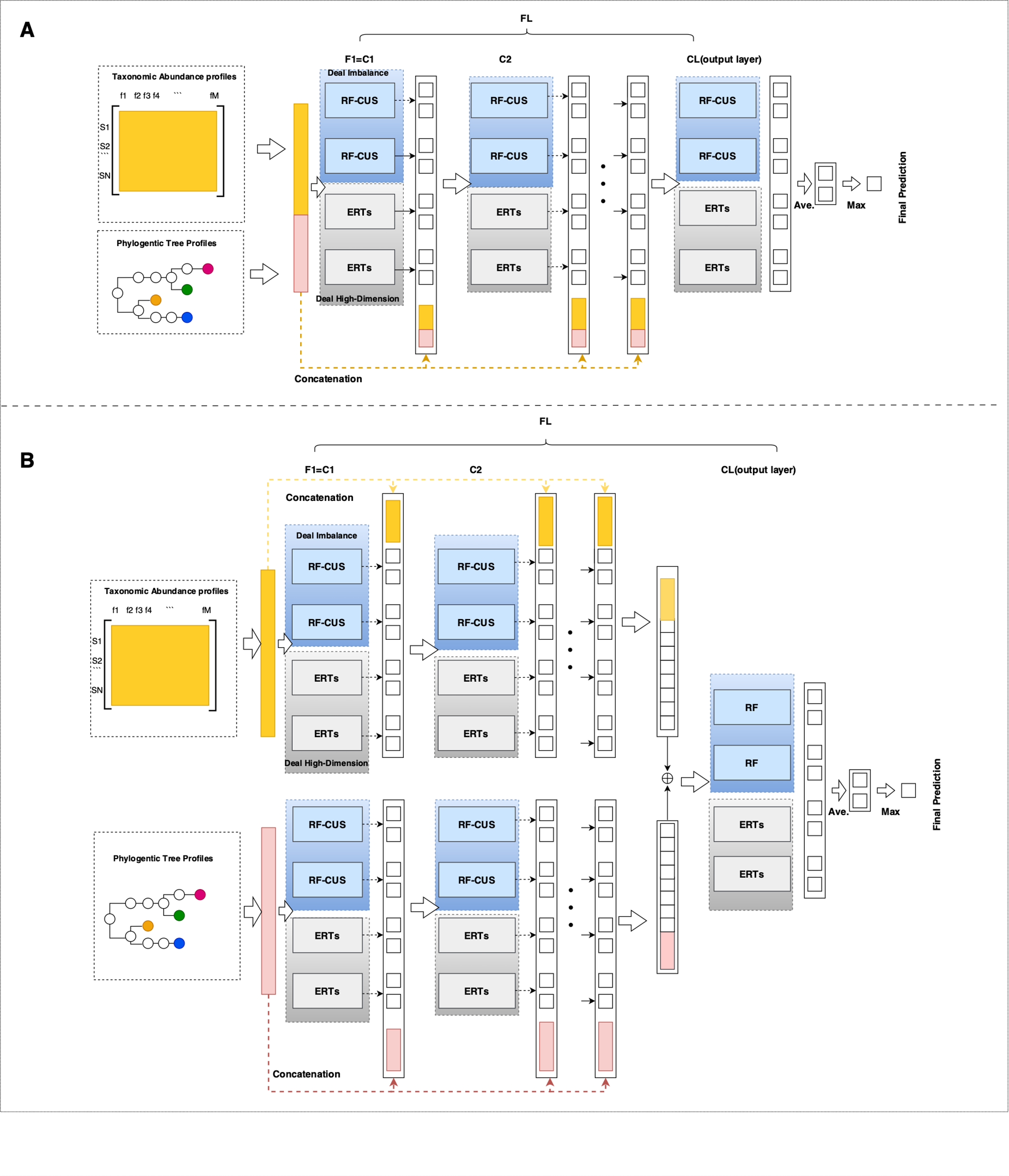


Figure S4. The schematic diagram of difference architecture learning from microbial data. A. Representing single-channel learning module utilised a modified deep forest model to simultaneously learn the microbial abundance profile and phylogenetic tree features. B. Representing two-channel learning module enabled independent learning of the embeddings of phylogenetic tree features and microbiological abundance features, with the learned embeddings from both channels combined as inputs to the prediction module.

Based on the Cirrhosis dataset, we compared several normalization methods for abundance data, including relative abundance(TSS), TSS + log, and TSS + CLR. Overall, normalization methods based on relative abundance achieved the highest performance across most metrics (Table S13).

Table S13 Performance Comparison of Different Normalization Methods on the Cirrhosis Dataset

| Normalization Method | tss | tss+log | tss+clr |
| --- | --- | --- | --- |
| auc | 0.9469 | 0.9272 | 0.9338 |
| aupr | 0.9480 | 0.9185 | 0.9240 |
| acc | 0.8993 | 0.8623 | 0.8624 |
| recall | 0.9126 | 0.9130 | 0.9130 |
| f1 | 0.8891 | 0.8669 | 0.8672 |

In our cross-cohort comparisons, we evaluated our model's performance on IBD disease data using various batch effect removal algorithms, including MMUPHin, Support Vector Decomposition (SVD), Remove Batch Effect (RBE), and Percentile Normalization (PN). While MMUPHin was not the most effective method in terms of performance, its requirement for non-negative input made it easier to compare with other models, as SVD, RBE, and PN can produce negative values(Table S14).

Table S14 Performance Comparison of Different Methods for batch effect removing on the IBD Dataset

|  | TS:IBD | | | TS:N_IBD | |
| --- | --- | --- | --- | --- | --- |
|  |  | auc | aupr | auc | aupr |
| TR:IBD | SVD |  |  | 0.6124 | 0.7806 |
|  | RBE |  |  | 0.6039 | 0.7962 |
|  | PN |  |  | 0.5842 | 0.7725 |
|  | MMUPHin |  |  | 0.5899 | 0.7252 |
| TR:N_IBD | SVD | 0.5631 | 0.7911 |  |  |
|  | RBE | 0.6174 | 0.8061 |  |  |
|  | PN | 0.6280 | 0.7638 |  |  |
|  | MMUPHin | 0.6273 | 0.7690 |  |  |
